# The radish genome and comprehensive gene expression profile of tuberous root formation and development

**DOI:** 10.1038/srep10835

**Published:** 2015-06-09

**Authors:** Yuki Mitsui, Michihiko Shimomura, Kenji Komatsu, Nobukazu Namiki, Mari Shibata-Hatta, Misaki Imai, Yuichi Katayose, Yoshiyuki Mukai, Hiroyuki Kanamori, Kanako Kurita, Tsutomu Kagami, Akihito Wakatsuki, Hajime Ohyanagi, Hiroshi Ikawa, Nobuhiro Minaka, Kunihiro Nakagawa, Yu Shiwa, Takuji Sasaki

**Affiliations:** 1 Tokyo University of Agriculture, 1-1-1, Sakuragaoka, Setagaya-ku, Tokyo 156-8502, Japan; 2 Mitsubishi Space Software Co., Ltd., 1-6-1, Takezono, Tsukuba, Ibaraki 305-0032, Japan; 3 Junior College of Tokyo University of Agriculture, 1-1-1, Sakuragaoka, Setagaya-ku, Tokyo 156-8502, Japan; 4 National Institute of Agrobiological Sciences, 1-2, Owashi, Tsukuba, Ibaraki, 305-8634, Japan; 5 Sakata Seed Corporation, 2-7-1, Nakamachidai, Tuzuki-ku, Yokohama, 224-0041, Japan; 6 National Institute for Agro-Environmental Science, 3-1-3, Tukuba, 305-8604, Japan

## Abstract

Understanding the processes that regulate plant sink formation and development at the molecular level will contribute to the areas of crop breeding, food production and plant evolutionary studies. We report the annotation and analysis of the draft genome sequence of the radish *Raphanus sativus* var. *hortensis* (long and thick root radish) and transcriptome analysis during root development. Based on the hybrid assembly approach of next-generation sequencing, a total of 383 Mb (N50 scaffold: 138.17 kb) of sequences of the radish genome was constructed containing 54,357 genes. Syntenic and phylogenetic analyses indicated that divergence between *Raphanus* and *Brassica* coincide with the time of whole genome triplication (WGT), suggesting that WGT triggered diversification of Brassiceae crop plants. Further transcriptome analysis showed that the gene functions and pathways related to carbohydrate metabolism were prominently activated in thickening roots, particularly in cell proliferating tissues. Notably, the expression levels of sucrose synthase 1 (SUS1) were correlated with root thickening rates. We also identified the genes involved in pungency synthesis and their transcription factors.

Radish (*Raphanus sativus* L.) is a diploid (2n = 2x = 18) dicot and an economically important root crop cultivated in worldwide. The thick roots and fresh sprouts are commonly harvested as vegetables, while some cultivars are used as leafy vegetables. In North America, radish seeds are produced for oil production, while siliques are also consumed in tropical Asia. Radish was used as food in ancient Egypt (2700–2200 bc) and has a long history of domestication (from 13^th^ century bc^1^). The wild ancestor (*Raphanus raphanistrum* L.), including several subspecies, is distributed mainly in the coastal regions from the Mediterranean to the Black Sea. Therefore, radish can grow in infertile soil and have a high tolerance to salinity and water deficit. Until now, numerous local and commercial varieties varying in root size, colour, contained materials and cropping types have been developed.

*Raphanus* belongs to the family Brassicaceae and tribe Brassiceae, which includes economically important crops such as *Brassica*; radish is closely related to *Brassica* crops. Within the Brassicaceae ancestral karyotype 24 genomic blocks were identified, and these 24 genomic blocks represent the conserved segments among the Brassicaceae ancestral karyotype^2^. Syntenic analysis between the *Brassica rapa* and *Arabidopsis thaliana* genome^3^ and comparative linkage mapping among some *Brassica* species and *A*. *thaliana*^4^,^5^ have suggested that genomes of the diploid *Brassica* species are triplicated and rearranged compared to the ancestral genome. These insights suggest that genome duplication may have triggered the evolution of *Brassica* crops. Comparative genomics among Brassiceae crops including *Raphanus* and other Brassicaceae species increase our understanding on the evolution of commercially important crop species.

The most remarkable characteristic of radish is its tuberous roots, which show huge variations in shape and size. The radish tuberous roots vary from a diameter of more than 30 cm to less than 1 cm, with a length from 3 cm to more than 2 m. The vegetative growth of radish is largely divided into four stages: germination, seedling, rosette and maturity. During the early seedling stage, root growth mostly occurs through elongation. About 10 days after germination (DAG), the true leaves appear and root tuberisation initiates, with the timing of tuber initiation differing slightly among cultivars. Until about 3 weeks to 1 month, the root slowly thickens (primary root thickening stage) and finally the primary cortex splits at the late seedling stage because the cortex cells cannot divide and expand. Cortex splitting is the sign of entry into the secondary root thickening stage mainly involving root tuberisation. Therefore, the radish tuberous root is an excellent model system for exploring mechanisms of plant sink formation and development.

The tuberous root contains high amounts of glucosinolates, the secondary metabolites found in the order Brassicales, whose hydrolysis products contribute to pungency, flavours and tastes. Specifically, pungency is one of the most important traits of radish cultivars. Compound 4-methylthio-3-butenyl isothiocyanate (4-MTBITC), the primary isothiocyanate responsible for the pungency of radish, is produced from aliphatic glucosinolate (4-methylthio-3-butenyl glucosinolate; 4-MTBGLS) through enzymatic hydrolysis by myrosinase (thioglucoside glucohydrolase)^6^,^7^. Isothiocyanate has high medical value as an alternative treatment for various alignments including hyperlipidaemia, coronary heart diseases and cancer^8^. The radish roots are also rich in carbohydrates, ascorbic acid, folic acid, potassium, vitamin B6, riboflavin, magnesium, copper and calcium. Despite its biological uniqueness and agricultural importance, molecular genetic studies of radish have been limited. Understanding the mechanism of radish root development should facilitate genetic improvement for radish yield, nutritional value and the quality of its appearance.

Recently, the draft genome of *R. sativus* var. *hortensis*, Japanese radish with long and thick roots, was published^9^. However, the lengths of generated scaffold and contig sequences are relatively small for applying gene structure and expression analyses. Here, we report the improved genome sequence of another Japanese radish strain assembled from 122-fold next-generation sequencing (NGS) data. Subsequently, we show the global transcriptome profile of sink (tuberous root) and source (leaf) organs at key developmental stages using RNA sequencing techniques based on genome sequence. The differentially expressed genes (DEGs) among root and leaf organs at several key developmental stages (7, 14, 20, 40, 60 and 90 DAG) are expected to be involved in the regulation of radish root organogenesis. Understanding the processes that regulate tuberous root formation and development at the molecular level will contribute to the areas of breeding, food production, medicinal care and plant evolutionary studies.

## Results

### Genome sequencing and assembly

With a total of 121.8× sequence data, the *R. sativus* genome was assembled using ≥500-bp and ≥2-kb scaffolds (Table 1). In total, 40,123 scaffolds ≥500 bp were generated spanning 383.3 Mb (N50: 138.17 kb, longest size: 1.31 Mb, average size: 9.55 kb), while 8686 scaffolds ≥2 kb were generated spanning 353.77 Mb (N50 158.63 Kb, longest size: 1.31 Mb, average size: 40.73 kb). The scaffolds ≥500 bp corresponded to 72,909 contigs with N50 of 7.12 kb, whereas the scaffolds ≥2 kb corresponded to 41,473 contigs with N50 of 8.15 kb. The quality of the scaffolds was evaluated by mapping onto the genome of 17,181 mRNA sequences from a wide variety of *R. sativus* cultivars (Supplementary Tables S1 and S2). The scaffolds and contigs were deposited at the DDBJ (DNA Data Bank of Japan) under accession numbers DF196826–DF236948 and BAOO01000001–BAOO01072909, respectively. We used flow cytometry to estimate the genome size of *R. sativus* (Supplementary Table S3), and the estimated 574 Mb genome size was similar to previous results by Johnston *et al*.^10^. Based on the estimated genome size, the ≥500-bp and ≥2-kb scaffolds covered approximately 67% and 63%, respectively, of the entire genome.

### Repetitive sequences

In total, 140.48 Mb repetitive sequences were detected for the scaffolds ≥500 bp in the radish genome (Table 2), and 4978 unique sequences were found in scaffolds ≥2 kb. The frequency and classes of repeat sequences in *R. sativus* (36.61%) were similar to that in *B. rapa* (39.51%).

### Gene models

In total, 64,657 *R. sativus* gene models were predicted (Table 1). Comparison with the gene models of *B. rapa*^3^ showed a shorter average gene locus length (1724 bp in radish and 2015 bp in *B. rapa*) and a longer mean exon length (266 bp in radish and 233 bp in *B. rapa*).

To complement the annotation of the genome sequence, gene models were mapped to the longest 53,846 open reading frame (ORF) sequences from the mRNAs of young leaves (Supplementary Table S4). In total, 3650 (6.8%) gene models had no ORF hit sequences, 22,949 gene models had more than 50% coverage, 9755 gene models had more than 90% coverage and 5554 gene models had 100% coverage (Supplementary Table S5).

In total, 54,132 gene models by BLASTp and 56,539 gene models by InterProScan have been characterised in terms of protein domain or functional sites, including 31,921 gene models with Gene Ontology (GO) classification. The paths of extracted GO IDs from the top node were searched and the classification of the 2^nd^ node of paths is shown in Supplementary Table S6 together with the results of similar analyses for *A. thaliana*^11^ and *B. rapa*^3^.

The numbers of specific and common gene families among *A. thaliana*^11^, *Arabidopsis lyrata*^12^ (excluding chloroplast and mitochondrial genes), *B. rapa*^3^, *Carica papaya*^13^ and *R. sativus* are shown as Venn diagrams in Supplementary Fig. S1. Among 200,700 protein sequences, 135,065 genes (67.3%) were clustered into 25,813 orthologous groups (Supplementary Table S7) and 6304 genes were clustered into 1272 *R. sativus*-specific clusters.

### Phylogenetic analysis

**Figure 1:**
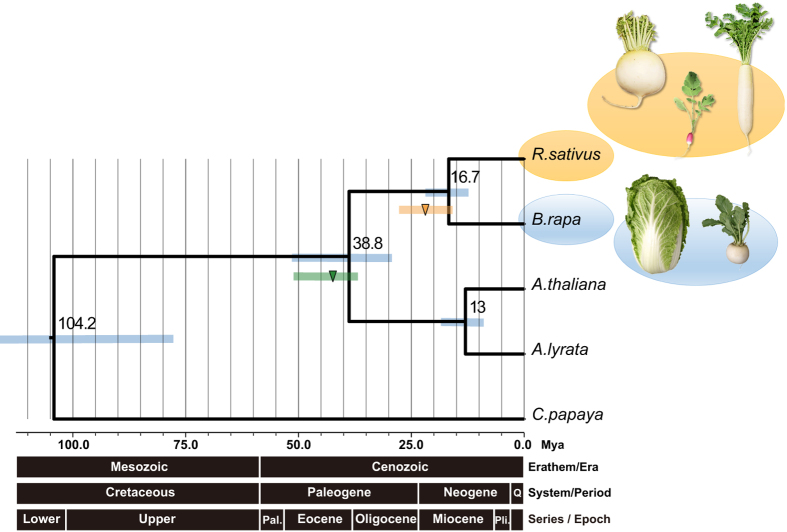
Phylogenetic tree of *R. sativus, A. thaliana, A. lyrata, B. rapa* and *C. papaya.* The blue line represents the 95% probability density (95PD) of ages for all nodes in the tree; the green line represents the most recent common ancestor (95PD: 50.7–43.2–36.6 Mya) of the *Arabidopsis–Brassica* clade; the orange line represents the whole genome triplication (95PD: 28.3–22.5–15.6 Mya) in the Brassiceae crown group (centre of the bold character is the average). The geologic timescale below the phylogenetic tree is based on the International Chronostratigraphic Chart (v2013). Photographs were taken by Y. Mitsui.

The lineages of *A. thaliana*, *A. lyrata* and related families diverged from a common ancestor at 5^13^,^14^, 10^3^ and 13^3^ million years ago (Mya), respectively, while the whole genome triplication (WGT) in *Brassica* occurred about 28.3–15.6 Mya^3^ and 5–9 Mya^3^. We applied OrthoMCL to the clustered and aligned gene models of *A. lyrata*, *A. thaliana*, *B. rapa*, *C. papaya* and *Oryza sativa* as reported by Wang *et al.^3^*, as well as *R. sativus*, and then selected a “single copy gene” set. Based on the lineage I phylogenetic divergence of *A. thaliana*, which occurred about 13 Mya, the divergence between *R. sativus* and *B. rapa* was estimated at 16.7 Mya (95PD: 22.4–12.4 Mya), much earlier than that between *A. thaliana* and *A. lyrata* around 13 Mya (95PD: 17.4–9.7 Mya; Fig. 1). The branch era between *Arabidopsis* and the *Brassica–Raphanus* clade was 38.8 Mya (95PD: 51.8–29.1 Mya), and the branch era between the *Brassica–Raphanus* clade and *C. papaya* was 104.2 Mya (95PD: 139.1–78.1 Mya; Supplementary Fig. S2).

### Genome structure

**Figure 2:**
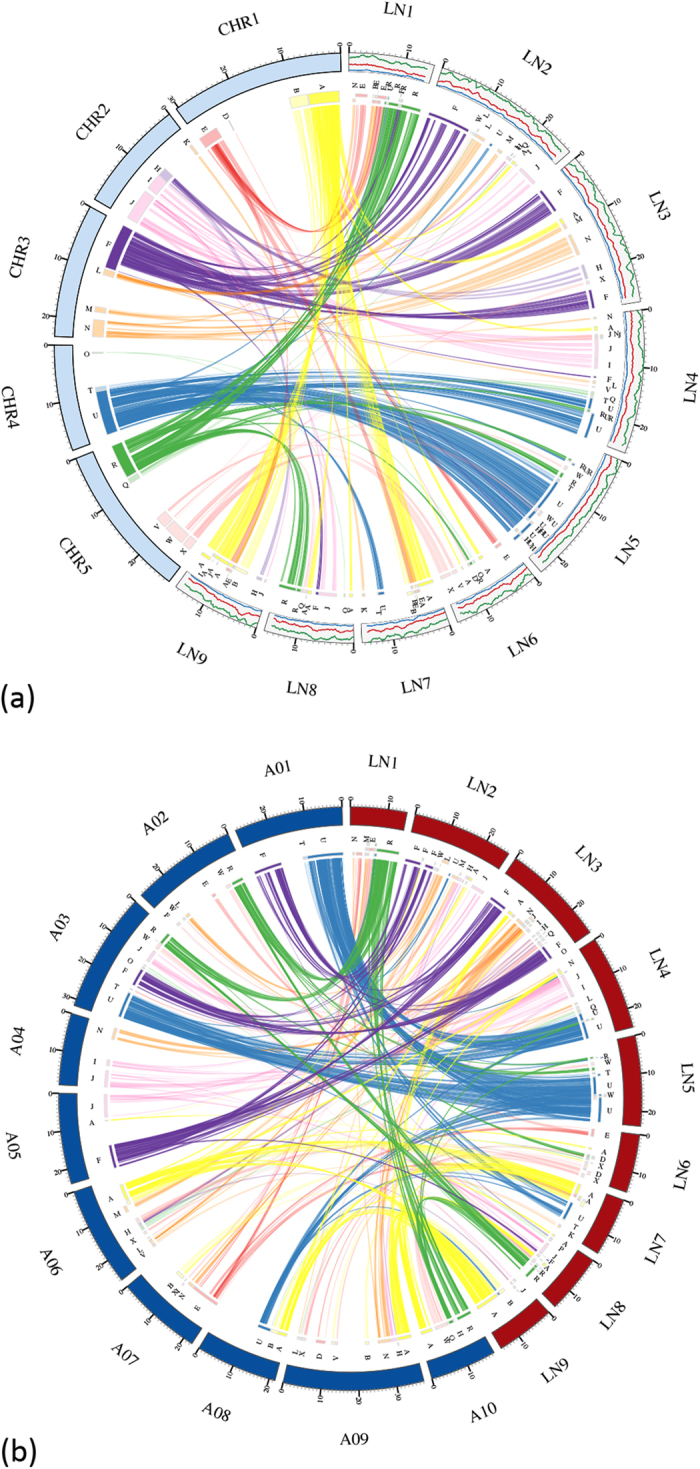
Syntenic relationships between *A. thaliana* and *R. sativus* (a) and between *B. rapa* and *R. sativus* (b) based on the ABC genomic blocks. The left arc represents *A. thaliana* and the right arc shows the line chart for repeat density. Red: retrotransposon, blue: DNA transposon, green: other repeat sequences.

In total, 1384 markers were mapped onto the pseudomolecules and spanned 179.8 Mb (137.2 Mb without gap) (Supplementary Table S8). The syntenic blocks for *R. sativus* vs. *A. thaliana* (Fig. 2a, Supplementary Fig. S3a), *R. sativus* vs. *B. rapa* (Fig. 2b, Supplementary Fig. S3b) and *R. sativus* vs. *R. sativus* (Supplementary Fig. 3c) were analysed using BLASTp and represented as “ABC” blocks^2^. The syntenic blocks for each linkage group are shown in Supplementary Fig. 4a and 4b. Based on the genome assembly, relatively large synteny blocks were observed between *A. thaliana* and *R. sativus*. Regarding the “A” block, large blocks were identified in LN7 and LN9 and small blocks in LN2, LN3, LN4, LN6 and LN8. Large “F” blocks exist in LN2 and LN3 (2 blocks) and small blocks in LN1 and LN8. Large “U” blocks exist in LN4, LN5 and LN7, and small blocks are found in LN1 and LN2. Large “R” blocks exist in LN1 and LN8 and small blocks in LN4, LN5 and LN6. “F” and “U” blocks had three synteny blocks, and the blocks were recognised from self-syntenic blocks. WGT has been observed in *B. rapa*^3^, and synteny analysis has shown that partial WGT may also have occurred in *R. sativus*.

### Radish Genome Database

All sequencing data can be accessed at http://www.nodai-genome-d.org/ with BLAST and GBrowse functions.

### RNA-seq and gene clustering analyses

**Figure 3:**
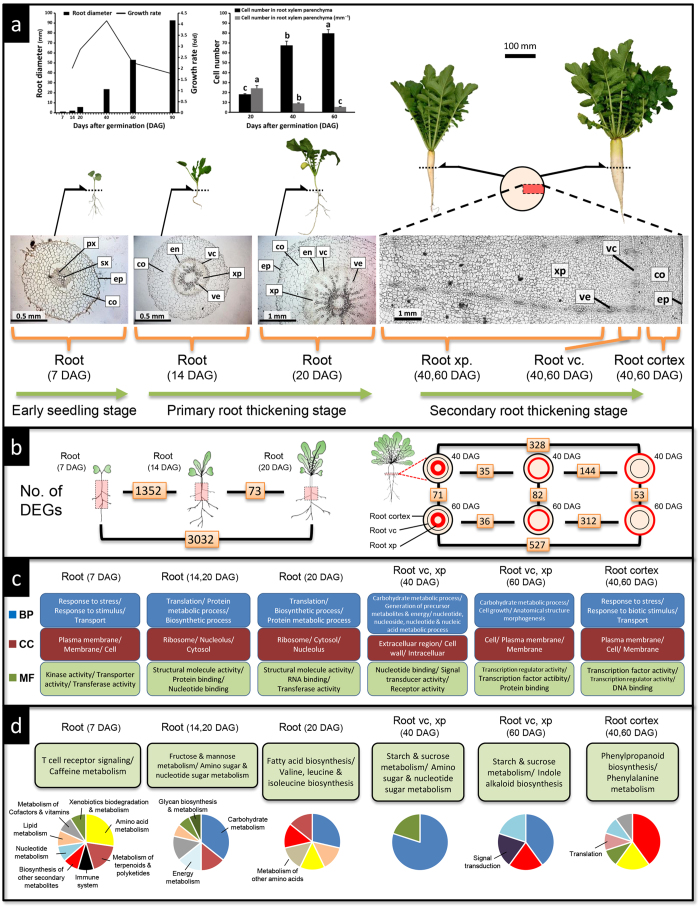
The process of root tuberisaiton of *R. sativus* var.*hortensis* cv. Aokubi and gene expression patterns at key developmental stages and tissues. (**a**) Root thickening and cell proliferating processes in 7, 14, 20, 40 and 60 days after germination (DAG) roots. Changes in root diameter, growth rate and cell number in xylem parenchyma tissue of 7–90 DAG roots are presented. Bar = SD. Letters represent significant differences among growing stages (ANOVA *post hoc* Tukey HSD, *P* < 0.01).Transverse section of roots are shown; px: primary xylem, sx: secondary xylem, co: root cortex, ep: epidermis, xp: xylem parenchyma, vc: root vascular cambium, en: endodermis, ve: vessel. (**b**) Number of differentially expressed genes (DEGs) in 7, 14, 20, 40 and 60 DAG roots and tissues. (**c**) Signifi**c**antly enriched GO categories for upregulated genes in each developmental stage and tissue. The top three categories of biological process (BP), cellular component (CC) and molecular function (MF) are shown. (**d**) Significantly enriched KEGG pathways for upregulated genes in each developmental stage and tissue. The top three pathways are shown. Photographs were taken by Y. Mitsui.

The process of radish tuberous root development and cell proliferating patterns are shown in Fig. 3a. Radish tuber initiated at about 14 DAG, and tuberisation occurred mainly by supplementation of proliferated cells from root vascular cambium (root vc) to xylem parenchyma (root xp) tissues. In root xp, enlargement of parenchyma cells was observed and cell proliferation occurred around vessels. Using the same radish strain used for genome sequencing, the global gene expression patterns in root and leaf tissues of key developmental stages were analysed. Using a single-end sequencing platform (Illumina, San Diego, CA, USA), an average of 2995 (SD: 645) million reads with 88% Q30 bases were generated from 14 cDNA libraries (Supplementary Table S9).

The majority of DEGs were found between early seedling roots (7 DAG roots) and the roots at the primary thickening stages (14 and 20 DAG roots), and also between cell proliferating tissues (root vc and root xp) and cortex tissue of 40 and 60 DAG roots (Fig. 3b). The data sets from early seedling to primary growth stage (7, 14 and 20 DAG roots) and from root vc, xp and cortex tissues in the secondary growth stage (40 and 60 DAG root tissues) were grouped together to detect DEGs. ANOVA tests detected 4260 and 4526 DEGs in 7, 14 and 20 DAG roots and 40 and 60 DAG root tissues, respectively. We also detected 10,468 DEGs in 7, 14, 20, 40 and 60 DAG leaf samples.

In each of the three data sets of the DEGs, cluster analysis was performed using self-organising maps (SOMs) to identify classes of genes with similar temporal changes. The DEGs of 7, 14 and 20 DAG roots, 1538 genes in 7 DAG roots (Cluster 6), 1035 genes in 14 and 20 DAG roots (Cluster 7) and 1539 genes in 20 DAG roots (Cluster 1) were clustered as overexpressed genes (Supplementary Fig. S5). In the DEGs of 40 and 60 DAG root tissues, the clusters of 877 genes in the 40 DAG root vc and xp (Cluster 7), 1187 genes in 60 DAG root vc and xp (Cluster 1) and 1498 genes in 40 and 60 DAG root cortex (Clusters 6 and 9) were detected (Supplementary Fig. S6). The thickening tissues (root vc and xp) showed similar gene expression patterns. In the DEGs of 7, 14, 20, 40 and 60 DAG leaves, we found that the gene clusters were associated with specific developmental stages (Supplementary Fig. S7).

### Gene functions and pathways involving tuberous root formation and development

Based on GO annotations, 40,705 of 65,457 *Raphanus* gene models were assigned GO terms. Statistical analysis revealed significantly enriched functional gene groups in each of the gene sets clustered as particular developmental stages and tissues (Fig. 3c, Supplementary Table S10). Genes related to stress and stimulus responses, transport and membrane activities were most significantly enriched in the cluster of genes upregulated at the early seedling stage of the root and leaf. In the cluster of genes upregulated in roots of the primary thickening stages, genes related to ribosomal activity, structural molecule activity and translation were most significantly enriched. In the cluster of genes upregulated in the root tissues (root vc and xp) of secondary thickening stages, genes related to membrane activities, transcription and cell development were particularly enriched, while genes related to stress and stimulus response, transport and membrane activities were most significantly enriched in the cluster of genes upregulated in root cortex tissue, similar to early seedling stage roots.

In total, 142 KEGG (Kyoto Encyclopaedia of Genes and Genomes) pathways including 13,795 genes were found in the *Raphanus* gene models. The significantly enriched KEGG pathways in each of the DEG clusters are shown in Fig. 3d and Supplementary Table S11. In thickening roots and cell proliferating tissues, the starch and sucrose metabolism pathway was particularly enriched. In the leaves in root thickening stages, pathways related to photosynthetic activities were activated. In root cortex tissue, the phenylpropanoid biosynthetic pathway that synthesises lignin was most significantly enriched.

Sucrose metabolism is considered important for the development of a plant sink organ. Thus, spatial and temporal changes in the expression of sucrose metabolism genes in radish sink and source tissues were analysed (Supplementary Fig. 8). The sucrose transporter genes (SUTs and SUCs) that function in cell-to-cell and long-distance distribution of sucrose throughout the plant showed relatively high expression rates in early seedling roots and leaves. The expression of genes encoding sucrose invertases, which function in sucrose cleavage, was relatively low. Among them, the cell wall invertase genes (CWIs) were expressed at a low level during all developmental stages. The expression of cytoplasmic invertase genes (CINs) increased only in the young seedling roots. We did not identify any vacuolar invertase genes (VINs) in the radish genome. Sucrose synthases (SUSs), the other enzymes involved in sucrose cleavage, showed markedly increased expression in tuberising roots and tissues. Two homologous genes of SUS1 were present and one showed particularly high expression in radish tuber, whereas these genes were expressed at low levels in the roots and leaves of early seedling stages. In the leaves of root thickening stages, the expression of SUS genes was limited. Additional RT-qPCR experiments confirmed that SUS1 genes were specifically expressed in tuberising roots and tissues and the expression levels of SUS1a were dozens of times higher than those in non-tuberising roots (Supplementary Fig. S9).

### Glucosinolate biosynthesis and myrosinase genes

**Figure 4:**
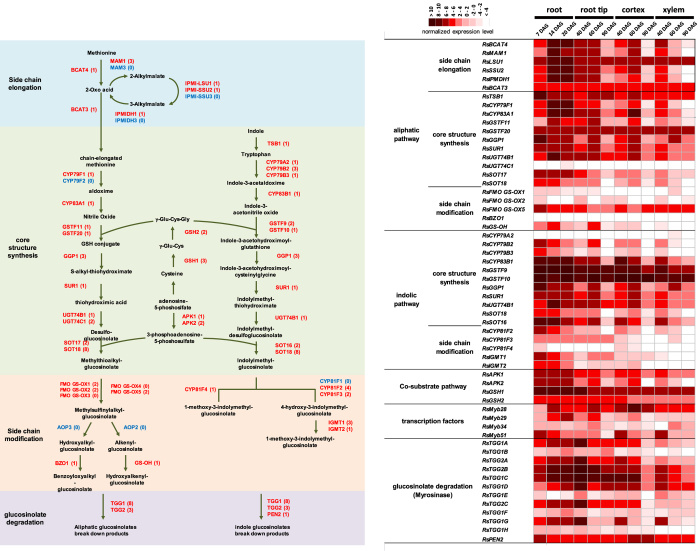
Glucosinolate biosynthesis and degradation genes in *R. sativus.* (**a**) Glucosinolate biosynthesis and degradation pathway. The number of genes in the genome is noted in brackets. Orthologues identified in *R. sativus* are marked in blue colour. The absence genes in the genome are marked in red. (**b**) Comparison of transcriptional profiles for glucosinolate biosynthesis and degradation genes. Heat maps show log2-scaled reads per kilobase per million reads (RPKM) for biosynthetic genes.

We investigated the genes involved in radish glucosinolate biosynthesis and degradation using radish genome information and transcriptome analysis. A core pathway of glucosinolates (GSLs) biosynthesis has been well examined and the genes have been identified in *A. thaliana*^16^,^17^. The *R. sativus* genome contained the majority of GLS biosynthesis-related genes (GLS genes), whereas no orthologue in the genome was observed for 10 GLS genes encoding amino acid side-chain elongation genes (MAM3, IPMI-SSU3 and IPMIDH3), core structure formation genes (CYP79F2), side-chain modification genes (CYP81F1, FMOGS-OX3, FMOGS-OX4, AOP2 and AOP3) and a transcription factor gene (MYB76) (Fig. 4, Supplementary Table S12). However, 8 genes are absent (IPMI-SSU3, IPMIDH3, CYP79F2, FMOGS-OX1, FMOGS-OX3, FMOGS-OX4, AOP3 and MYB76) in the related *B. rapa* genome^16^. Thus, genes that are not contained in both genomes may be lost in ancestors of *R. sativus* and *B. rapa*, or may be specific to *Arabidopsis* and its relatives.

In radish roots, the tip and outer zone including peel are known to be a pungent region, and isothiocyanates and glucosinolates are dominantly detected in this region^19^,^20^. Our transcriptional profiling has shown that numerous GSL genes were strongly expressed in root tip, cortex and vc corresponding to the pungent region in the root (Fig. 4b and Supplementary Fig. S10). Moreover, GSL genes were expressed in younger developmental stages (14 and 20 DAG), which generally showed a high accumulation of GSLs^20^ (Fig. 4b and Supplementary Fig. S11). In this manner, GSL gene expression showed spatial and temporal correlations with GSL accumulation and pungency. These results indicated that the expression of GSL genes plays an important role in GSL accumulation and pungency, and that their expression was coordinately regulated in radish roots. MYB28, MYB29, MYB76, and R2R3-type MYB family transcription factors have been reported to regulate the expression level of GSL genes in *A. thaliana*^21^,^22^. Based on transcriptome profiles, expression of the radish gene encoding a protein homologous to the *Arabidopsis* MYB29 protein showed correlations with GSL gene expression (Fig. 4b and Supplementary Fig. S12) and MYB28 showed relatively high expression in pungent tissues (Fig. 4b). These results indicate that the core glucosinolate biosynthesis pathway and the regulation mechanisms are conserved in Brassicales.

Eleven myrosinase encoding genes were identified in the radish genome. The number of myrosinase genes is distinctly large in the Brassicae and *Raphanus* genus in the order Brassicales. The myrosinase genes are also expressed in pungent regions of the root and middle developmental stages, and were correlated with the presence of isothiocyanates (Fig. 4b and Supplementary Figs S13, S14). These findings demonstrated that transcriptional regulation of glucosinolate biosynthesis and myrosinase genes is important for determining the pungency level in radish roots.

Additional RT-qPCR experiments of four genes, MYB28, BCAT4, CYP79F1 and TGG1-3 (TGG1C), showed that these pungency-related genes were highly expressed in pungent tissues (Supplementary Fig. S15).

## Discussion

We constructed a draft genome sequence of *R. sativus*. Generated scaffolds (≥500 bp) showed improved values for N50 (138.17 kb; longest size, 1.31 Mb; average size, 9.55 kb) compared to previously published radish genome assemblies (scaffolds >300 bp; N50, 46.26 kb; longest size, 0.831 Mb); average size, 5.25 kb^9^. The total length of scaffolds assigned to the linkage map was 179.8 Mb, much longer than in previous reports (116.0 Mb)^9^. The number of predicted gene models was comparable between the two studies.

Divergence between *R. sativus* and *B. rapa* was estimated to be about 16.7 Mya (95PD: 22.4–12.4 Mya). WGT has been suggested to have occurred at around 27.3–15.6 Mya in the ancestral lineage of tribe Brassiceae based on analyses using fossil records^23^. Our results indicated that WGT may play a key role in the Brassiceae, which includes commercially important vegetable crops. Syntenic analysis between *R. sativus* and *B. rapa* revealed that duplicated regions within each genome were different. Previous studies also reported that the genome synteny between these species is complicated^24^,^25^, suggesting that extensive genome rearrangements took place during or after the divergence between *Raphanus* and *Brassica*. These rearrangements may have occurred through the process of chromosome reduction from the ancestral karyotype^26^. When comparing phylogenetic trees and chronostratigraphic charts, branch eras between *Arabidopsis* and the *Brasssica–Raphanus* clade corresponded to the *Eocene* period, and the branch era between *Raphanus* and *Brasssica* corresponded to early parts of the *Miocene* period. Both periods were climatic warming periods^27^,^28^, suggesting that global warming may accelerate the divergence.

Transcriptomic analyses revealed the global gene expression profile of radish tuber initiation and development. The DEGs were mostly found between roots during the early seedling stage and roots at the primary thickening stages. During the early seedling stage (until about 10 DAG), radish roots rapidly elongate to more than 50 cm (Y. Mitsui, unpublished data) and then enter the thickening stages. In roots at the primary thickening stage and cell proliferating tissues at the secondary root thickening stage, genes involved in translation and several metabolic pathways were similarly activated, suggesting that gene activation may switch from root elongation to tuberisation, and that similar gene expression systems would continue to promote root tuberisation. Meanwhile, stress and stimulus response, transport and signalling gene activities were highly activated in early seedling roots and in cortex tissues of thickening roots. As early seedling roots are largely composed of cortex tissues, these tissues likely play a central role in transport and signalling activities in radish roots. Moreover, the phenylpropanoid biosynthetic pathway synthesising lignin was mostly activated in the cortex tissues of thickening roots, suggesting that biosynthesis and accumulation of lignin in the cortex contributes to maintaining the strength of radish tubers.

We found that sucrose metabolism pathways were most significantly activated in radish tuberising roots. Sucrose cleavage is vital to multicellular plants for carbon metabolism and plant sink development. The reactions of invertase and sucrose synthase catalyse sucrose cleavage^29^. Genes encoding the cell wall and cytoplasmic and vacuolar invertases showed low expression in developing roots, while some CINs were activated in the seedling stage roots and leaves. Invertases are believed to mediate the initiation and expansion of new sink structures^30^,^31^. CINs are generally less active compared with the other invertases, but our result suggests that CINs play a role in radish sink initiation.

The expression of SUSs was strongly associated with the root thickening rate, suggesting that SUSs play a central role in sucrose metabolism for tuber development, which is consistent with previous studies showing that shifts from invertase to sucrose synthase paths of sucrose cleavage could occur in later developmental phases and facilitate storage and maturation of sinks^32^,^33^. SUS is a key enzyme involved in carbohydrate metabolism that catalyses the reversible conversion of sucrose and uridine diphosphate (UDP) into fructose and UDP-glucose. In sink tissues, the UDP-glucose product of sucrose synthase has been implicated in the formation of starch and in the synthesis of callose and diverse cell wall polysaccharides^34^,^35^. In *Arabidopsis*, a multigene family encodes six SUS isoforms (SUS1–6). Two isoforms of SUS have been reported in radish^36^. We found that all six unigene homologues (excluding SUS4) are present in the radish gene models. Among them, only SUS1 was exclusively expressed in enlarging roots. SUS plays a crucial role in the development of sweet potato^37^ and potato^38^,^39^ tubers, and also the tomato fruit^40^. These results are suggestive of a common mechanism for the biochemical pathways in the development of plant sink organs.

In the early seedling roots and leaves, genes encoding sucrose/H+ symporters (SUTs or SUCs) were specifically expressed; proton-driven sucrose transporters play a crucial role in cell-to-cell and long-distance distribution of sucrose throughout the plant^41^. Membrane-localised SUTs or SUCs mediate the loading of sucrose into the phloem in source tissues^41^, and thus concentrations of sucrose in the conducting vascular cells reach hundred-fold higher levels than in source cells^42^,^43^. Sucrose/H+ symporters are also localised in sink tissues and are thought to be involved in unloading sucrose into the apoplast^44^,^45^. In the early seedlings of radish, some sucrose transporters promote loading and unloading of sucrose. As root tuberisation begins, SUS activity markedly increases in sink tissues, suggesting that a switch in the sucrose metabolic pathway occurs during tuber initiation. High levels of SUS enzymes involved in the breakdown of sucrose increase sink capacity by lowering the local concentration of sucrose, thereby generating a gradient that allows further unloading of sucrose from the phloem^46^,^47^. We found that the pathway of carbon fixation in photosynthetic activities was significantly activated in the leaves of root tuberising stages, suggesting that high amounts of sucrose are supplied from source leaves into phloem, generating the hydrostatic pressure difference between source and sink tissues that drives the mass flow of water and nutrients in the phloem vessels. A previous report showed that CO_2_ elevation resulted in an enhanced sink capacity of radish that was responsible for absorbing higher levels of photosynthates^48^, and the activity of SUS was dependent on the amount of carbohydrate supply from the source organs^29^,^49^. Thus, the level of photosynthetic activity should be positively correlated with the root growth rate. Overall, our results indicate that spatio-temporal gene expression of some sucrose transporters, invertase and sucrose synthases in source, sink and phloem tissues play a critical role in sucrose metabolism, resulting in tuberous root initiation and subsequent development.

The glucosinolate–myrosinase system found in the Brassicales is a well-studied plant secondary metabolism. Molecular biological studies on glucosinolate biosynthesis have been conducted with a focus on the model plant *Arabidopsis*, but it was insufficient in agricultural species, including radish^17^. Characterisation of the radish glucosinolate biosynthesis-related genes and transcriptome analysis have provided further evidence that expressional regulation of their GLS genes is critical in determining the pungency level in radish roots. R2R3-type MYB transcription factors are known transcriptional regulators of aliphatic glucosinolates biosynthesis^21^,^22^, and the radish genome encodes multiple copies of the R2R3-type MYB transcription factors homologous to MYB28 and MYB29. Based on transcriptional analysis, the radish MYB28 and MYB29-like gene showed dominant expression in pungent stages and parts of the root, and the expression was correlated with GLS gene expression patterns. This finding indicates that MYB28 and MYB29-like genes act as a key regulator of glucosinolate biosynthesis in radish roots. Furthermore, these findings suggest that GSL biosynthesis regulation mechanisms are evolutionally conserved in the Brassicaceae. In contrast, differences in the composition of the MYB gene, such as the absence of MYB76, was observed between *R. sativus* and *A. thaliana*.

The composition of accumulated glucosinolates shows differences among the Brassicales^50^. The dominant glucosinolate in radish is 4MTB-GSL, classified as a short-chain aliphatic glucosinolate. The gene composition of GSL biosynthesis-related genes and their expression plays an important role in determining species-specific glucosinolates profiles. Although GLS genes were mostly conserved among the Brassicales, especially between *R. sativus* and *B. rapa*, radish-specific gene composition was observed in the genome. MAM1 and MAM3 genes encode methylthioalkylmalate synthase, which is involved in the methionine side-chain elongation step in glucosinolate biosynthesis^51^. MAM1 catalyses condensations in the first three elongation cycles, and MAM3 controls the aliphatic glucosinolate chain lengths in *Arabidopsis*^52^. Three MAM1-like genes were identified in the radish genome, whereas MAM3-like genes were absent. Furthermore, the AOP2 gene catalyses the oxidation of aliphatic glucosinolate, which is involved in side-chain modification and present in the radish genome. These differentiations of GLS gene compositions may be involved in radish-specific glucosinolate profiles and 4MTB-GSL accumulation.

Although the two radish myrosinase genes have been identified previously, the number of myrosinase genes in the radish genome and their expression patterns remain unclear^53^. Sequencing of the radish genome and global expression analysis have provided detailed expression profiles of myrosinase genes. The *R. sativus* and *B. rapa* genomes contain 11 myrosinase genes classified in the myrosinase MA, MB and MC subfamilies. The number of myrosinase genes in these two species is significantly higher than based on whole-genome sequences of other Brassicaceae. Therefore, an increased number of myrosinase genes was observed in ancestors of these species. Transcriptome analysis revealed differentiation of expression patterns among the 11 myrosinase genes, and 5 genes were mainly expressed in the root (Supplementary Fig. S16). Thus, the increased number and diversification of myrosinase genes, as well as their expression patterns, may be involved in protecting the root as a vegetative storage organ, as well as the formation of characteristic pungent roots. Myrosinase genes are expressed in the peel and root tip, as well as in the xylem in pungent cultivars^54^, which indicates that expression changes in myrosinase genes are involved in variations of pungency among radish subspecies.

For radish breeding, pungent traits have focused only on the development of condiment cultivars. Recently, isothiocyanates have been shown to induce apoptosis in various cancer cells, and the functionality has received attention from industry and acadaemia^55^. Radish genome information increases our understanding of the isothiocyanate production mechanism, which may be useful for the development of new valuable cultivars with functionality.

## Methods

### Estimation of genome size

The relative genome size of *R. sativus* was analysed using flow cytometry. The leaf samples were chopped finely using a razor blade and incubated on a petri dish containing extraction buffer. After 3 minutes, the resulting extract was passed through a CellTrics filter with 30-μm mesh. For propidium iodide (PI) staining of nuclear DNA, the CyStain PI plant DNA absolute quantitation reagent KIT05-5022 (Partec Inc., Franklin Park, IL, USA) was added to four times its volume and incubated for more than 1 hour before measurement. The relative fluorescence of total DNA was measured using the Partec CyFlow ploidy analyser (Green laser). Genomic DNA extracted from *A. thaliana* was similarly analysed using flow cytometry for comparison. Genome size was estimated based on the *pro rata* allocation between the median peak position of *A. thaliana* and *R. sativus*.

### Sequencing and assembly

Genome sequencing was performed using *R. sativus* var. *hortensis* cv. Aokubi doubled haploid (DH) line provided by Sakata Seed Co. (Yokohama, Japan). High-quality nuclear DNA with reduced organellar DNA was extracted from leaves of 20-day-old seedlings using a protocol modified from Paterson *et al*.^56^. We prepared approximately 70-Gb sequences including paired end (PE) sequences with insert lengths of 300 bp and 500 bp using the Illumina Hiseq 2000 at the Nodai Genome Research Centre, Tokyo University of Agriculture. Long-jumping-distance (LJD) library sequences with insert lengths of 8 kb, 20 kb and 40 kb were generated using the Illumina Hiseq 2000 at Operon Biotechnologies, Inc. (Huntsville, AL, USA). In addition, about 5-Gb single end sequences (SE) were analysed using the Roche 454 FLX Titanium at the Agrogenomics Research Centre, NIAS (National Institute of Agrobiological Sciences). Flow cytometry analysis resulted in 574 Mb corresponding to 122× genome coverage (Supplementary Table S13). The sequence data were deposited at the DDBJ (DNA Data Bank of Japan) as BioProject ID PRJDB707.

The SE, PE and 8-kb insert LJD sequence reads were filtered for duplicated sequences, trimmed by Quality Value (QV) and assembled separately using Newbler (GS-Assembler version 2.7, Roche/454, Branford, CT, USA), Ray (version 2.0.0)^57^ and CLC genomics workbench (version 5.5.1, CLC bio, Aarhus, Denmark). Based on subsequent evaluation of the results from these assemblers (Supplementary Table S14), we adopted the genome assembly generated by Newbler. Moreover, we removed organelle sequences from more than 500-bp contigs by BLASTn using the *B. rapa* (JF920285.1) and *R. sativus* (AB694744.1) mitochondrial sequences, and *B. rapa* (DQ231548.1) and *A. thaliana* (AP000423.1) chloroplast sequences were used as queries. The vector sequences were checked by BLASTn with pBACe3.6 (U80929.2) as the query. Subsequently, using the contigs and mate-pair (MP) sequences, scaffold sequences were built using SSPACE (version 2.0)^58^ (Supplementary Table S14).

### Evaluation of scaffolds

The quality of the scaffolds was evaluated by mapping a total of 17,181 mRNA sequences of *R. sativus* available at the NCBI (National Centre for Biotechnology Information) UniGene (http://www.ncbi.nlm.nih.gov/unigene) onto the genome using BLAT^59^.

### Repetitive sequences

Scaffolds >2 kb were analysed using REPET^60^,^61^. The copy number of repeat sequences for scaffolds >500 bp was determined using RepeatMasker (http://www.repeatmasker.org/) (Supplementary Table S15).

### Gene prediction and annotation

Gene models were generated using the gene prediction programme Augustus^62^ with *Arabidopsis* parameters. To complement the annotation of the genome sequence, we constructed a full-length cDNA library under the same conditions as the genomic DNA used for sequencing. mRNAs were assembled using Trinity^63^ to build contigs. The longest 53,846 ORF sequences were extracted from the contigs using EMBOSS^64^.

The gene models were processed by BLASTp^65^ with the NCBI nr database and by InterProScan^66^. The paths of extracted GO IDs from the top node were searched, along with the classification of the 2^nd^ node together with the results of similar analysis for *A. thaliana*^11^ and *B. rapa*^3^. The numbers of specific and common gene families among *A. thaliana*, *A. lyrata*^12^ (excluding chloroplast and mitochondrial genes), *B. rapa*, *C. papaya*^12^ and *R. sativus* are shown as a Venn diagram.

### Orthologous genes

Using the CD-HIT^67^ with -c 0.9 -n 5, we removed highly similar paralogous genes. After a BLASTp run with an all-against-all option, the BLAST results were fed into the stand alone OrthoMCL^68^ programme using a default MCL inflation parameter of 1.5.

### Phylogenetic analysis

We clustered the gene models of *A. lyrata*, *A. thaliana*, *B. rapa*, *C. papaya*, *O. sativa* and *R. sativus* using OrthoMCL (inflation factor 1.5) and selected “single copy gene” sets. The gene sets that perfectly matched with gene models assembled from the *R. sativus* transcriptome were selected. The branch era of each gene set was estimated using Baseml^69^ (HKY85, ncatG = 5) and Multidivtime^70^. Before estimation of branch era, a topology of the evolutionary tree was prespecified using Clustal W2^71^ and MrBayes^72^ as required when using Multidivtime. To clarify the overall pattern of selected gene sets, four-fold degenerate sites were extracted for the 720 gene sets, and each gene set was aligned using Clustal Omega^73^.

The scatterplot (Supplementary Fig. S2) shows a normal distribution with minimal bias. We checked specific genes in the clustered gene set with the annotated GO of *A. thaliana*^74^, but could not confirm specific genes. Based on these results, we constructed a phylogenetic tree using all 720 gene sets. Due to limitations of the Multidivtime programme, each of the 30 gene sets was aligned using Clustal Omega, and 24 aligned sequences were drawn using Mutidivtime. In Fig. 2, we adopted the calibration time of 13 Mya for the *A. thaliana*–*A. lyrata* clade^23^.

### Analysis of linkage groups

We constructed a linkage map with a total of 1384 markers (Supplementary Table S16) including 630 markers from the Kazusa DNA laboratory^75^, 746 markers from Tohoku University^25^ and 8 markers from Chonnam National University^76^. If the assigned linkage number and direction differed from the Kazusa and Tohoku linkage maps, we used the Kazusa linkage map information, which was published previously^75^. Both markers were coordinated with *A. thaliana* (Supplementary Table S17).

The linkage map was used for mapping scaffolds onto linkage groups. The Kazusa markers, which consist of expressed sequence tags (ESTs) and primer sequences, were used to search for EST sequences and scaffolds by BLAT^59^. The Tohoku markers consist of primers and probes. We searched for these three sequences and scaffolds using BLAST, and 446 markers were selected with three whole sets and one error. The Korea markers consist of ESTs and primer sequences. We searched for EST sequences and/or primer sequences and scaffolds using BLAST. These three sets of markers were merged, and 1039 markers (including derivative markers) were mapped onto the scaffolds.

### Syntenic analysis

The homology between *A. thaliana* and *R. sativus* was analysed using BLASTp with an e-value 1.0e-5. After filtering BLAST results using MCScan version 0.8^77^ with Match_Size 40, the syntenic relationship was analysed using the Circos viewer version circus-0.63-4^78^. A similar analysis was performed for *B. rapa* vs. *R. sativus* and *R. sativus* vs. *R. sativus*.

### Preparing RNA-seq samples

Seeds of the Japanese radish cultivar ‘Aokubi’ used in the genome study were sown in the experimental field of Tokyo University of Agriculture, Faculty of Agriculture, at the normal sowing time in this region (31 August 2012). Samples of roots and leaves from three seedlings at each developmental stage (7, 14, 20, 40 and 60 DAG) were collected. In radish tuberous roots, the upper part originates from the hypocotyl where lateral roots are not present and the lower part consists of true root tissue where lateral roots developed. We collected samples from the border of hypocotyl and true root tissues and immediately transferred the samples into RNAlater® solution (Qiagen, Hilden, Germany) for RNA extraction.

Total RNA was isolated from each sample using the NucleoSpin RNA Plant Kit (Macherey-Nagel, Düren, Germany). RNA quality and quantity were assessed on a 2100 Bioanalyser using the RNA 6000 Nano Kit (Agilent technologies, Palo Alto, CA, USA). Using equal volumes of total RNA from each sample, cDNA libraries were prepared using an mRNA-Seq Sample Preparation Kit (Illumina) according to the manufacturer's instructions. Illumina RNA sequencing using the Hiseq 2000 platform was performed at the Nodai Genome Research Centre (NRGC, Tokyo, Japan) in accordance with the manufacturer’s instructions.

### RNA-seq analysis

RNA-seq raw reads were imported into the CLC genomic workbench, and the reads were trimmed using default parameters. The 64,657 Augustus gene model^66^ was used as a reference sequence for mapping. The trimmed reads sets obtained from the cDNA libraries were mapped to reference sequences using default parameters. The reads mapped as paired reads were used for calculating gene expression values. The expression level for each transcript was calculated the using the reads per kilobase per million reads (RPKM) method. The BLAST2GO suite was used for GO and KEGG pathway analysis.

### Detecting differentially expressed genes and cluster analysis

DEGS were detected between all pairs of libraries using a *t*-test and among the libraries grouping key developmental stages and tissues by ANOVA using a Benjamini and Hochberg false discovery rate (FDR)-adjusted significance level of <0.05, a fold change of >2 and a minimum RPKM difference of >10. DEGs were clustered using a SOM algorithm and Genesis software (http://genome.tugraz.at/genesisclient/genesisclient_description.shtml). A Euclidean distance metric was used and nine (3 × 3) clusters were generated, which were visually inspected for similarity and differences among the gene expression profiles. Clusters were merged if they shared a similar expression profile.

### Gene ontology and pathway analyses

The curated clusters of genes from SOM analysis were further investigated and mapped to significant ontologies and pathways to analyse their biological function. The Blast2GO programme was used to obtain GO annotation of the unigenes based on BLASTp hits against the NCBI Nr database with an e-value threshold of <10^−5^. Each cluster of annotated genes was mapped to the KEGG pathway database and the gene number was calculated for every KEGG pathway. Then, Fisher’s exact test was used to match the significantly enriched KEGG pathway in the DEG clusters to the genomic background.

### RT-qPCR

To validate some candidate genes involved in sucrose metabolism and pungency biosynthesis, RT-qPCR experiments were conducted using LightCycler® 480 SYBR Green I Master (Roche) according to the manufacturer’s instructions, with 1 μM primers in a 10 μL final volume. Primer information is presented in Supplementary Table S18. Conditions for the amplification were as follows: a 10 min incubation at 95 °C, followed by 45 cycles (95 °C for 10 s; 60 °C for 10 s; 72 °C for 10 s) with a single fluorescent reading taken at the end of each cycle. All the runs were completed with a melt curve analysis to confirm the specificity of amplification and lack of primer dimers. Cp (second derivative method) values were used to estimate expression levels. The expression levels were adjusted by two housekeeping genes (Actin^79^ and Calmodulin 7:CAM7).

## Additional Information

**How to cite this article**: Mitsui, Y. *et al*. The radish genome and comprehensive gene expression profile of tuberous root formation and development. *Sci. Rep*. **5**, 10835; doi: 10.1038/srep10835 (2015).

## Supplementary Material

Supplementary Information

Supplementary Table S10

Supplementary Table S11

Supplementary Table S16

## Figures and Tables

**Table 1:** Assembly and annotation of the *R. sativus* genome.

	Scaffold ≥ 500b	Scaffold ≥ 2 Kb
**Assembly features**		
Number of scaffolds	40,123	8,686
Total span	383.30 Mb	353.77 Mb
N50 (scaffolds)	138.17 Kb	158.63 Kb
Average scaffold size	9.55 Kb	40.73 Kb
Longest scaffold size	1.31 Mb	1.31 Mb
Number of contigs	72,909	41,473
Longest contig	83.77 Kb	83.77 Kb
N50 (contigs)	7.12 Kb	8.15 Kb
GC content	40%	40%
**Gene models**		
Number of gene models	64,657	
Mean gene locus length	1,723.81 bp	
Mean coding sequence length	1,112.33 bp	
Mean number of exons per gene	4.18	
Mean exon length	266.37 bp	
Mean intron length	118.18 bp	

**Table 2:** Repeat sequences of *R. sativus* and *B. rapa*.

	*R.sativus*		*B.rapa*	
TE_subclass	Length (Mb)	Length/Scaffold (%)	Length (Mb)	Length/Scaffold (%)
DNA	14.16	3.70	15.52	3.20
LINE	15.92	4.16	13.69	2.82
LTR	41.65	10.87	131.61	27.14
SINE	2.62	0.68	2.24	0.46
Other	10.09	2.64	0.00	–
noCat	56.04	14.63	28.57	5.89
Total	140.48	36.68	191.63	39.51
