# Supplementary material for: The radish genome and comprehensive gene expression profile of tuberous root formation and development
Source: Sci Rep. 2015 Jun 9;5:10835. doi: 10.1038/srep10835 (PMC4650646; doi:10.1038/srep10835)
Supplement: Supplementary Information [file srep10835-s1.pdf]

The radish genome and comprehensive gene expression profile of tuberous root formation and development

Yuki Mitsui[1*], Michihiko Shimomura[2*], Kenji Komatsu[1*], Nobukazu Namiki[2], Mari Shibata-Hatta[1], Misaki Imai[1], Yuichi Katayose[3], Yoshiyuki Mukai[3], Hiroyuki Kanamori[3], Kanako Kurita[3], Tsutomu Kagami[4], Akihito Wakatsuki[4], Hajime Ohyanagi[2], Hiroshi Ikawa[2], Nobuhiro Minaka[1,5], Kunihiro Nakagawa[1], Yu Shiwa[1], Takuji Sasaki[1,3]

[1] Tokyo University of Agriculture, 1-1-1, Sakuragaoka, Setagaya-ku, Tokyo 156-8502, Japan

[2] Mitsubishi Space Software Co.,Ltd., 1-6-1, Takezono, Tsukuba, Ibaraki, 305-0032, Japan

[3] National Institute of Agricultural Sciences, 1-2, Owashi, Tsukuba, Ibaraki, 305-8634, Japan

[4] Sakata Seed Corporation, 2-7-1, Nakamachidai, Tuzuki-ku, Yokohama, 224-0041, Japan

[5] National Institute of Agro-Environmental Sciences, 3-1-3, Tukuba, 305-8604, Japan

* To whom correspondences should be addressed. Yuki Mitsui, Tel: +81 46 270 6289; Fax: +81 270 6289; Email: y3mitsui@nodai.ac.jp, Michihoko Shimomura, Tel: +81 29 859 0311; Fax: +81 29 859 0321; Email: Shimomura.Michihiko@mss.co.jp, Kenji Komatsu, Tel: +81 3 5477 2468; Fax: +81 3 5477 2628; Email: k4komats@nodai.ac.jp.

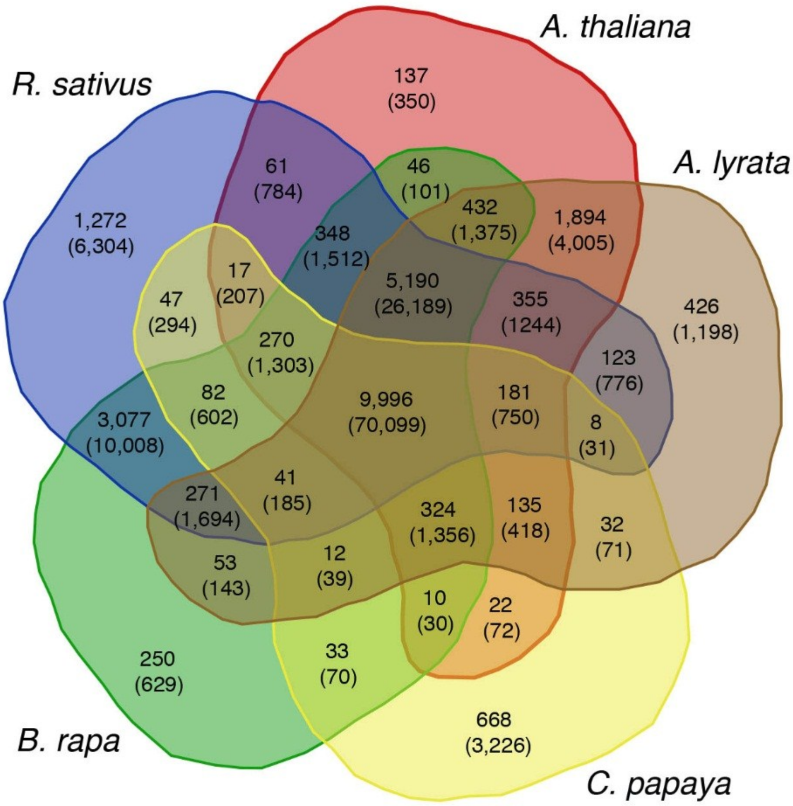

Supplementary Figure S1. Venn diagram of specific and shared gene families of *R. sativus* with related Brassicales species, namely, *A. thaliana, A. lyrata, B. rapa* and *C. papaya*, using Calculate and draw custom Venn diagrams (http://bioinformatics.psb.ugent.be/webtools/Venn/). Numbers in parentheses indicated the number of genes in each cluster. The gene families were obtained from the following data repositories: The Arabidopsis Information Resource (ftp://ftp.arabidopsis.org/home/tair/Genes/); Phytozome: *Arabidopsis lyrata* (ftp://ftp.jgi-psf.org/pub/compgen/phytozome/v9.0/Alyrata/); Brassica Database (http://brassicadb.org/brad/); and Phytozome: Carica papaya (ftp://ftp.jgi-psf.org/pub/compgen/phytozome/v9.0/Cpapaya/).

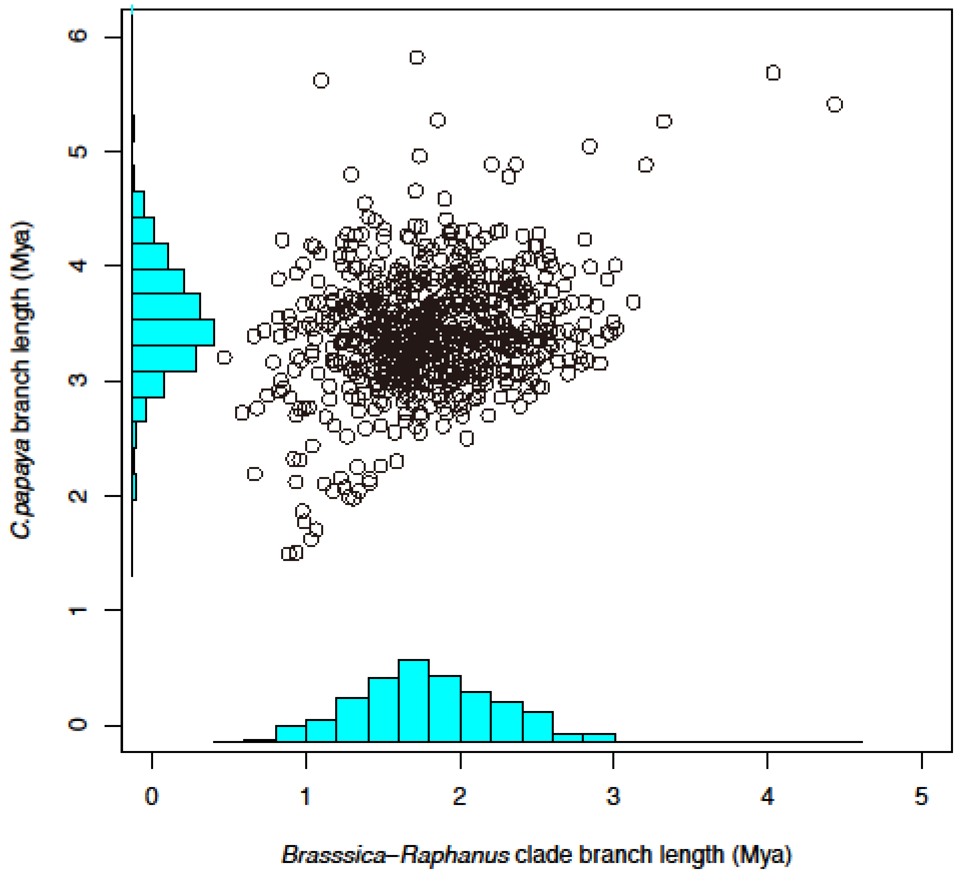

Supplementary Figure S2. Branch era distribution of *Brasssica-Raphanus* clade and *C. papaya*. The horizontal axis corresponds to the branch era of *A. lyrata, A. thaliana, B. rapa, R. sativus* clade (*Brasssica-Raphanus* clade) and the vertical axis corresponds to the branch era of *C. papaya*. Bar charts were normalized histogram of each axes.

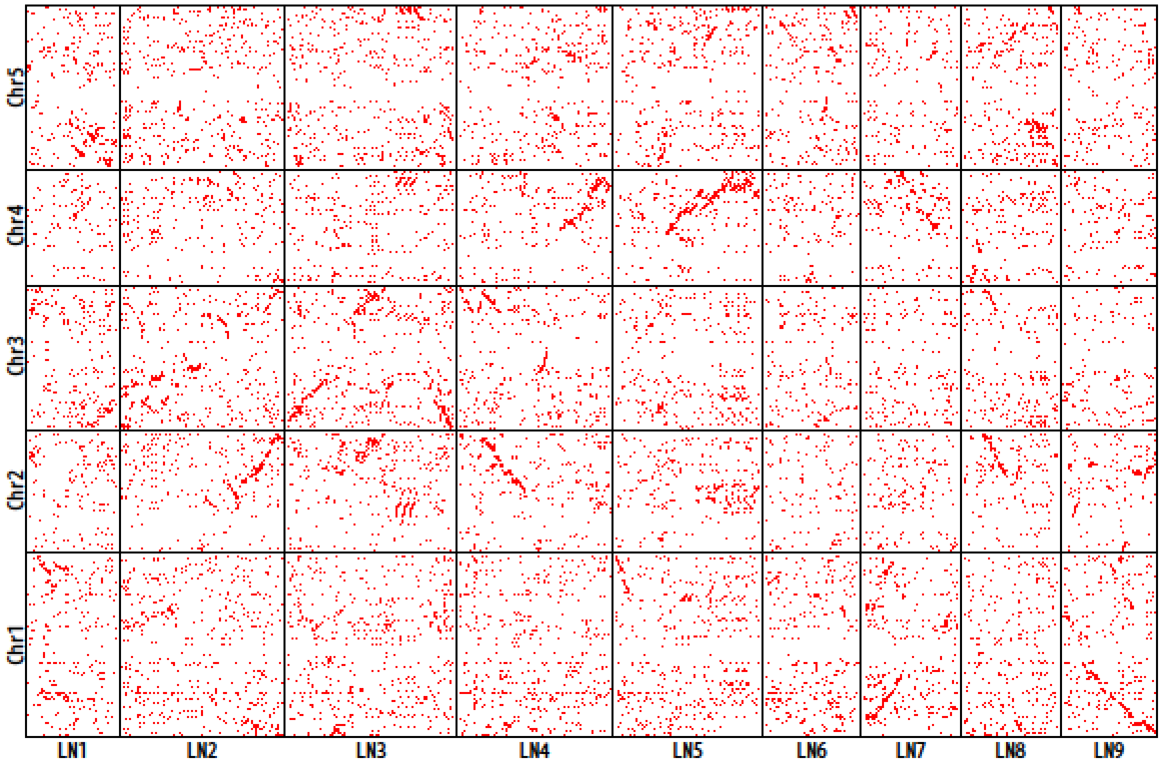

(a)

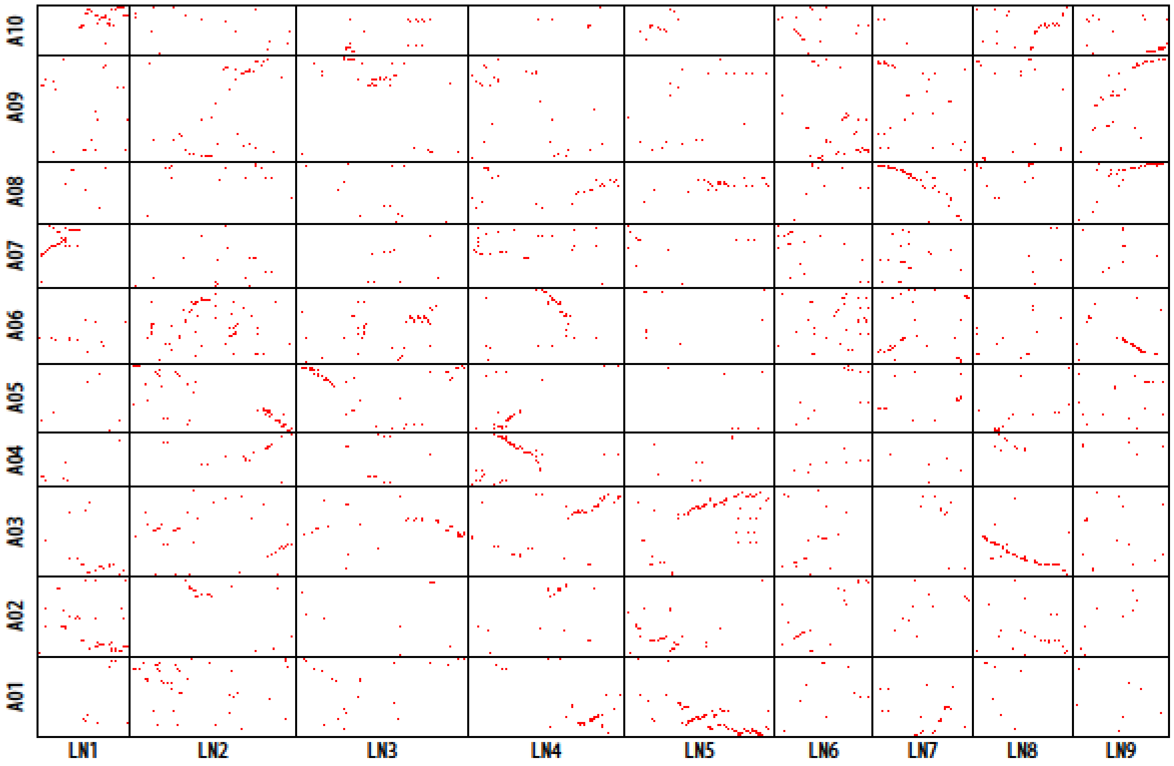
A10
A09
A08
A07
A06
A05
A04
A03
A02
A01
LN1
LN2
LN3
LN4
LN5
LN6
LN7
LN8
LN9

(b)

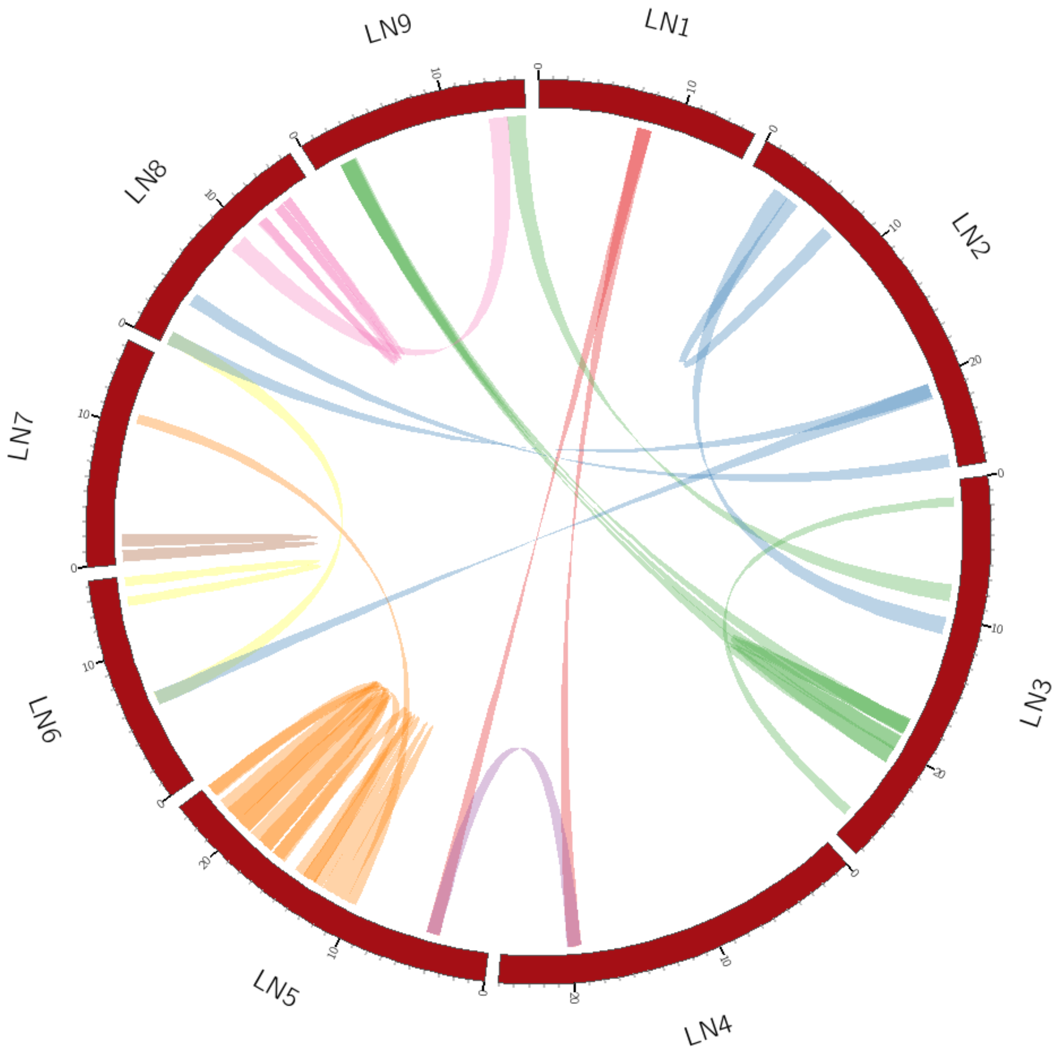

(c)

Supplementary Figure S3. Synteny blocks between *A. thaliana* vs. *R. sativus* (a), *B. rapa* vs. *R. sativus* (b) and *R. sativus* vs *R. sativus* (c) based on BLASTp e-value of 1.0e-5.

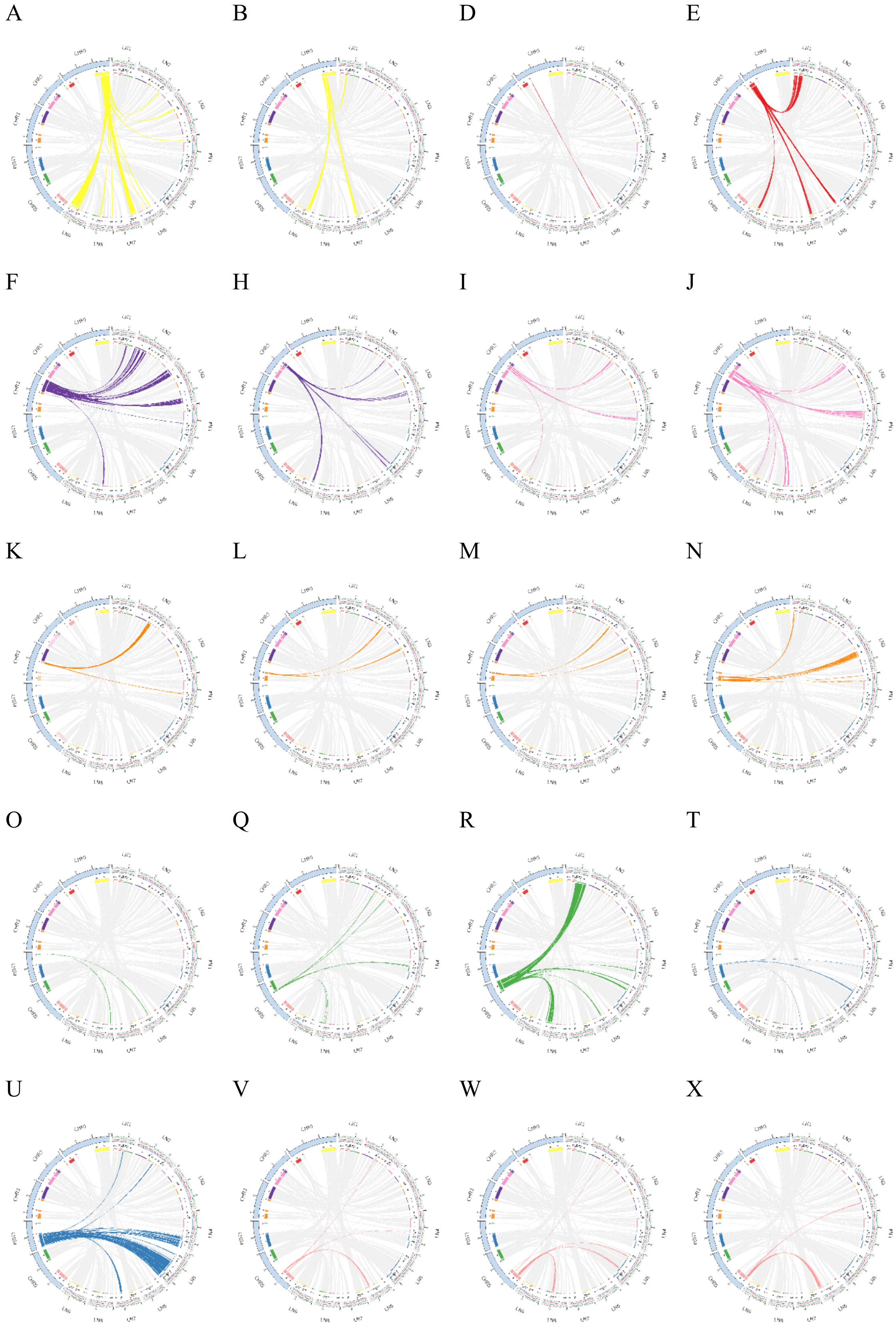

(a)

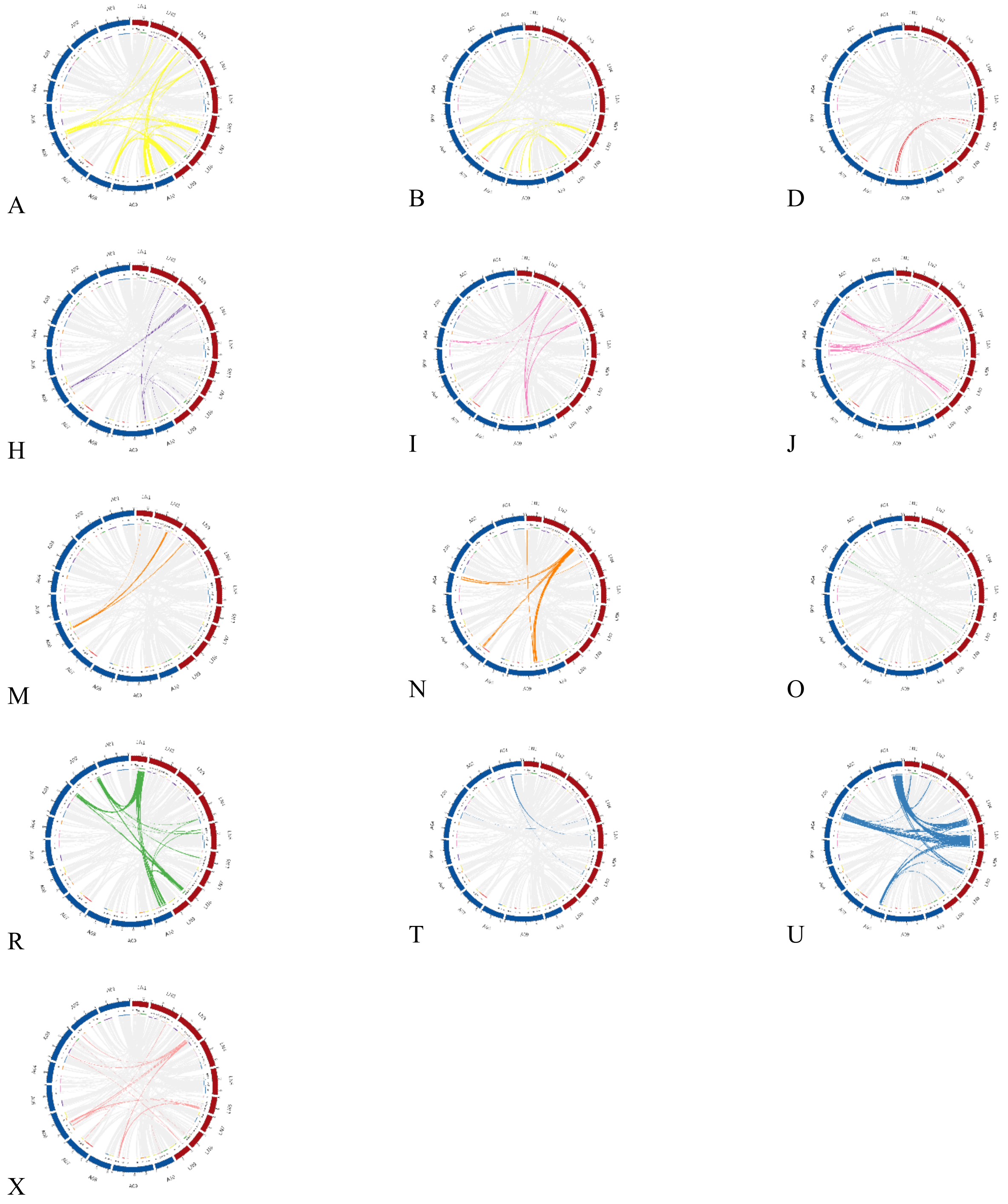

(b)

Supplementary Figure S4. Synteny between *A. thaliana* vs. *R. sativus* (a) and *B. rapa* vs. *R. sativus* (b) based on ABC blocks.

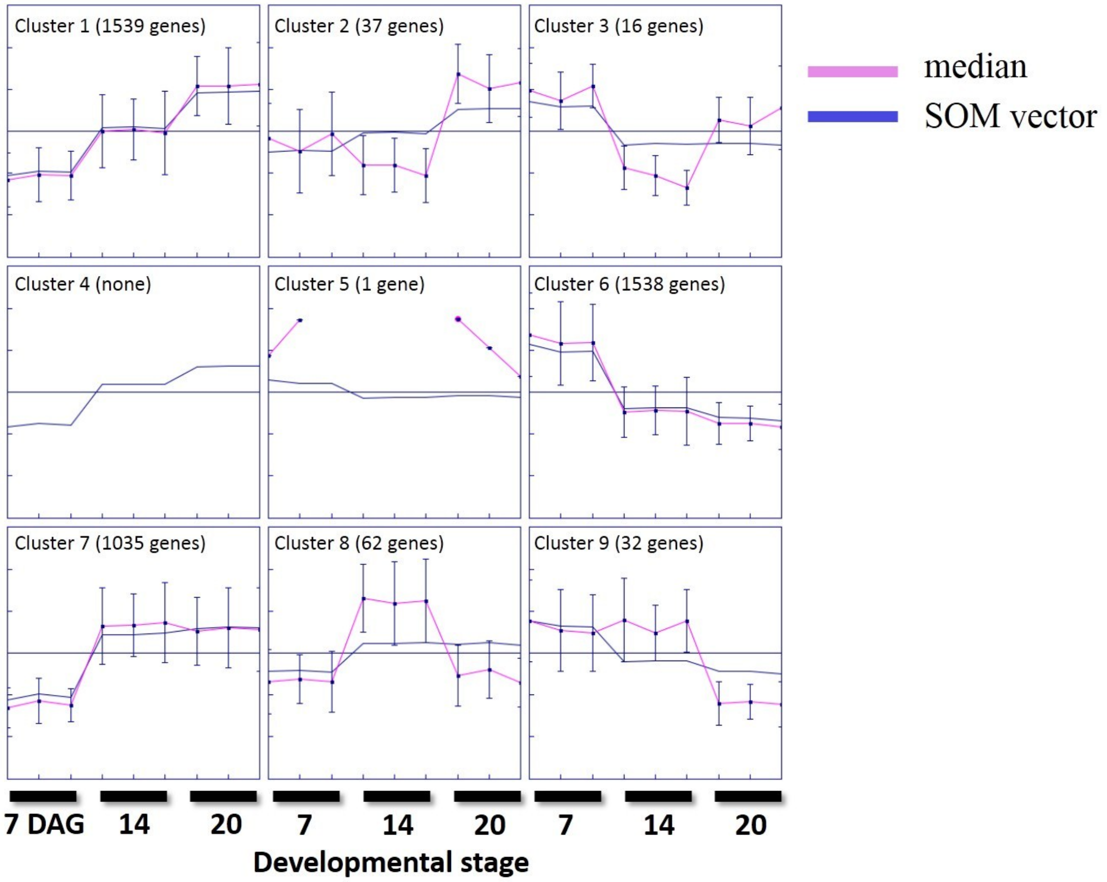

Supplementary Figure S5. Clustering analysis by self-organizing maps (SOM) in the 4,260 differentially expressed genes (DEGs) detected among 7, 14, 20 DAG roots.

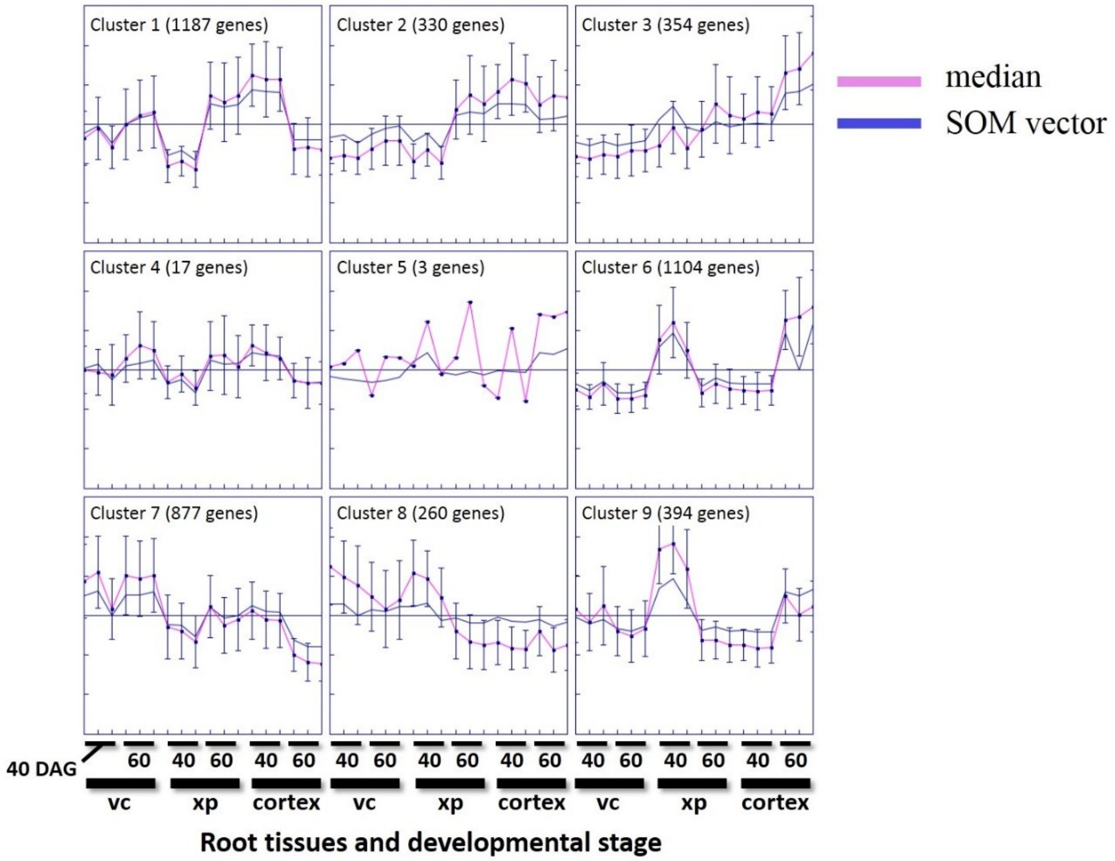

Supplementary Figure S6. Clustering analysis by self-organizing maps (SOM) in the 4,526 differentially expressed genes (DEGs) detected among 40 and 60 DAG root vascular cambium (vc), root xylem parenchyma (xp) and root cortex.

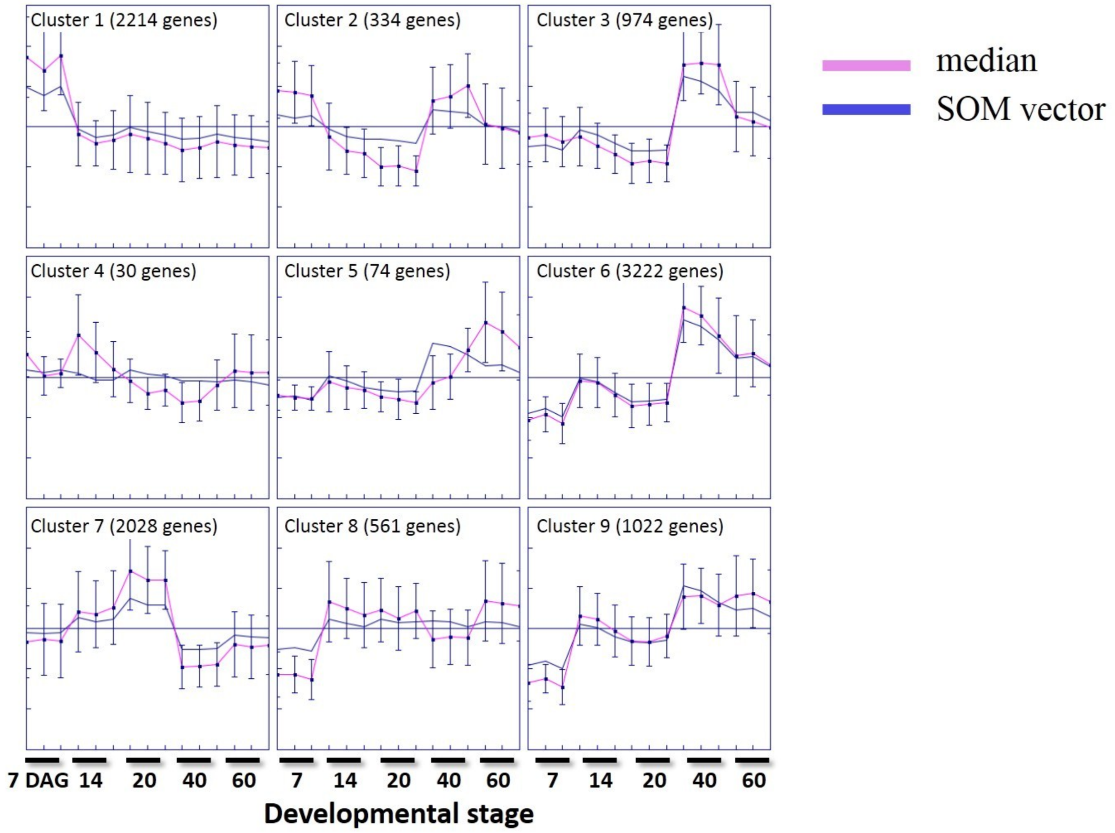

Supplementary Figure S7. Clustering analysis by self-organizing maps (SOM) in the 10,468 differentially expressed genes (DEGs) detected among 7, 14, 20, 40, 60 DAG leaves.

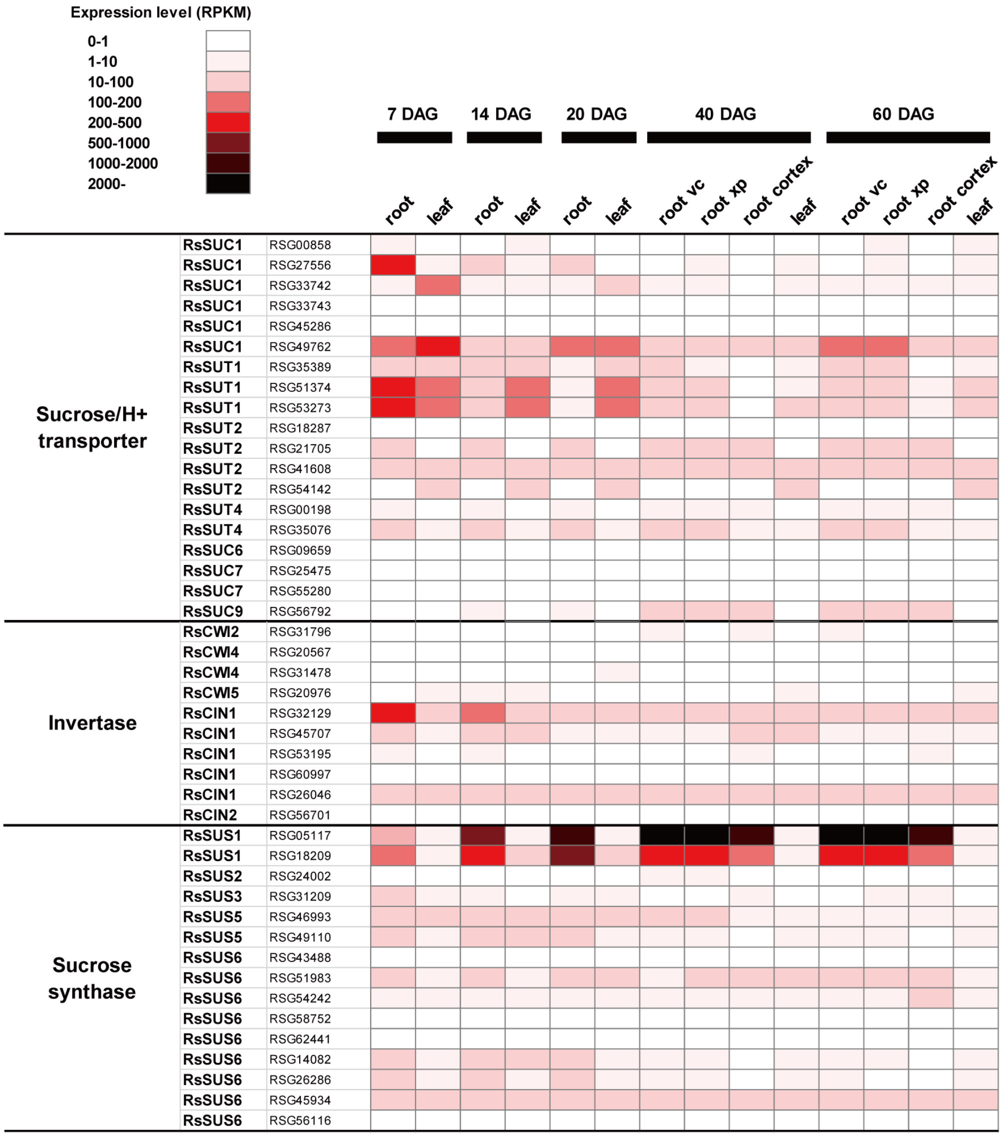

Supplementary Figure S8. Expression of genes encoding sucrose metabolism in multiple developmental stages and tissues.

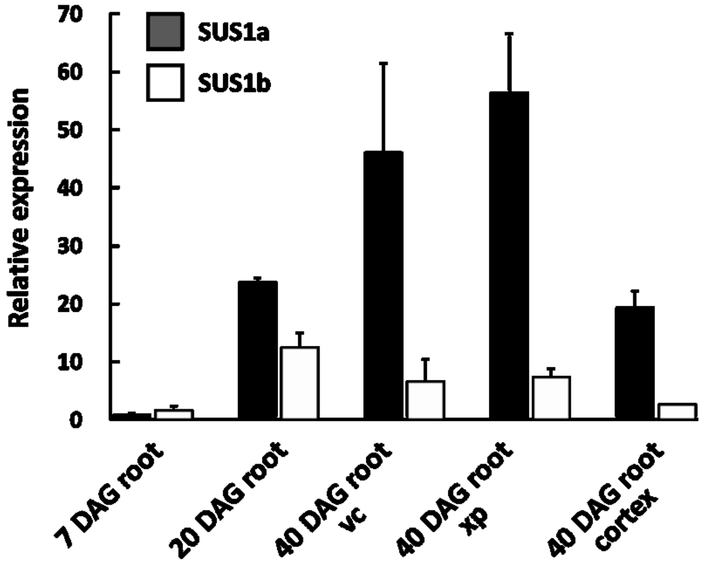

Supplementary Figure S9. The relative expression level of SUS1a (RSG05117) and SUS1b (RSG18209) based on RT-qPCR. The expression levels were adjusted by two reference genes (Actin and CAM7). The expression values of the two SUS1 genes are relative to those of 7 DAG roots. The values are mean ± SD (n = 3).

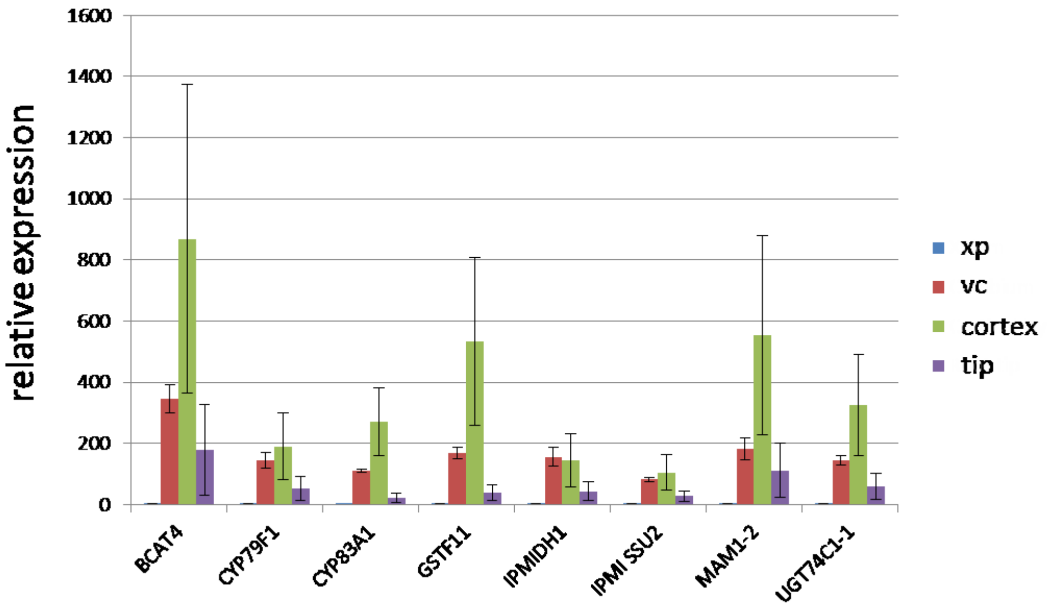

Supplementary Figure S10. The relative expression level of glucosinolate biosynthesis genes in root. The expression levels in root xylem parenchyma (xp), root vascular cambium (vc), root cortex and root tip were analyzed by RNA-seq analysis. The expression level of BCAT4, CYP79F1, CYP83A1, GSTF11, IPMIDH1, IPMI SSU2, MAM1-2 and UGT74C1-1 relative to those of the xylem parenchyma are shown. The values are mean ± SE (n = 3).

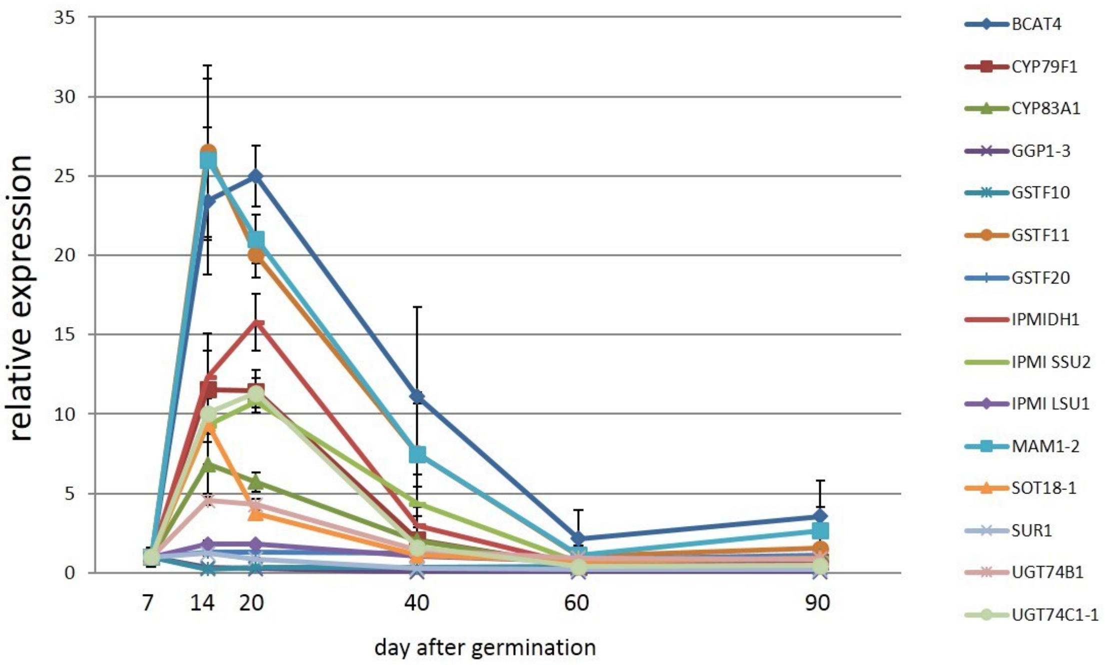

Supplementary Figure S11. The relative expression level of glucosinolate biosynthesis genes in root development. The expression levels in root (7, 14 and 20 days after germination: DAG) and root tip (40, 60 and 90 DAG) were analyzed by RNA-seq analysis. The expression level of BCAT4, CYP79F1, CYP83A1, GGP1-3, GSTF10, GSTF11, GSTF20, IPMIDH1, IPMI SSU2, IPMI LSU1, MAM1-2, SOT18-1, SUR1, UGT74B1 and UGT74C1-1 relative to those of the 7 DAG are shown. The values are mean ± SE (n = 3).

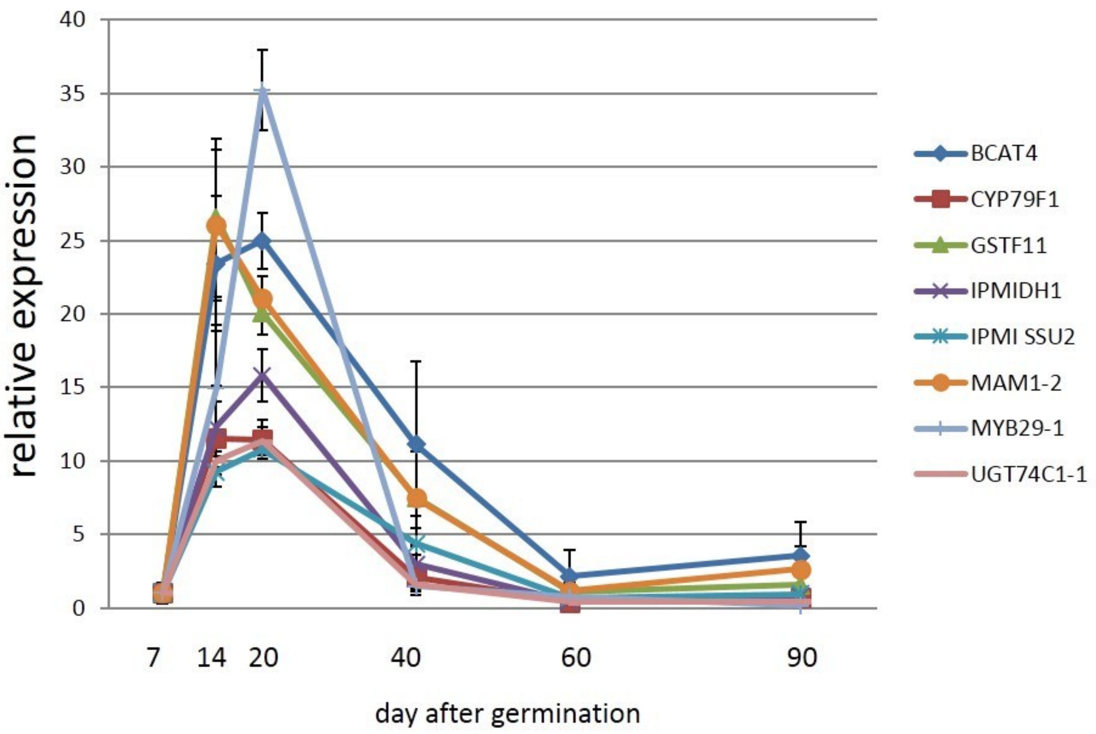

Supplementary Figure S12. The relative expression level of MYB29 and glucosinolate biosynthesis genes in root development. The expression levels in root (7, 14 and 20 days after germination: DAG) and root tip (40, 60 and 90 DAG) were analyzed by RNA-seq analysis. The expression level of BCAT4, CYP79F1, GSTF11, IPMIDH1, IPMI SSU2, MAM1-2, MYB29-1 and UGT74C1-1 relative to those of the 7 DAG are shown. The values are mean ± SE (n = 3).

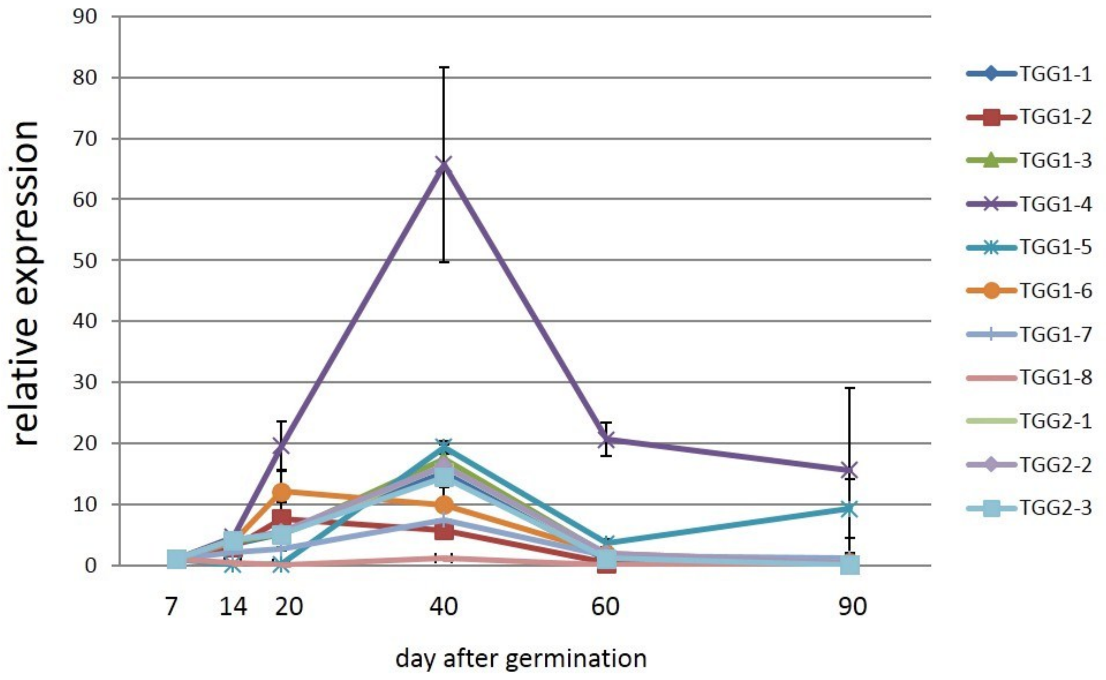

Supplementary Figure S13. The relative expression level of myrosinase genes in root development. The expression levels in root (7, 14 and 20 days after germination: DAG) and root tip (40, 60 and 90 DAG) were analyzed by RNA-seq analysis. The expression level of TGG1 and TGG2 like radish myrosinase genes relative to those of the 7 DAG are shown. The values are mean ± SE (n = 3).

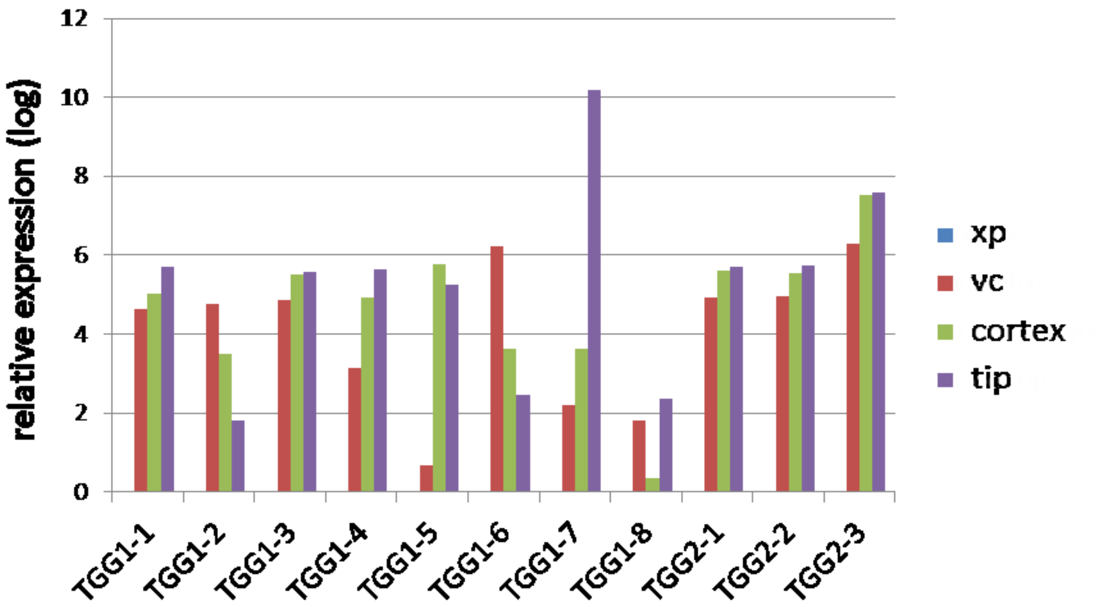

Supplementary Figure S14. The relative expression level of myrosinase genes in root. The expression levels in root xylem parenchyma (xp), root vascular cambium (vc), root cortex and root tip were analyzed by RNA-seq analysis. The expression level of TGG1 and TGG2 like radish myrosinase genes relative to those of the xylem parenchyma are shown. The values are mean ± SE (n = 3).

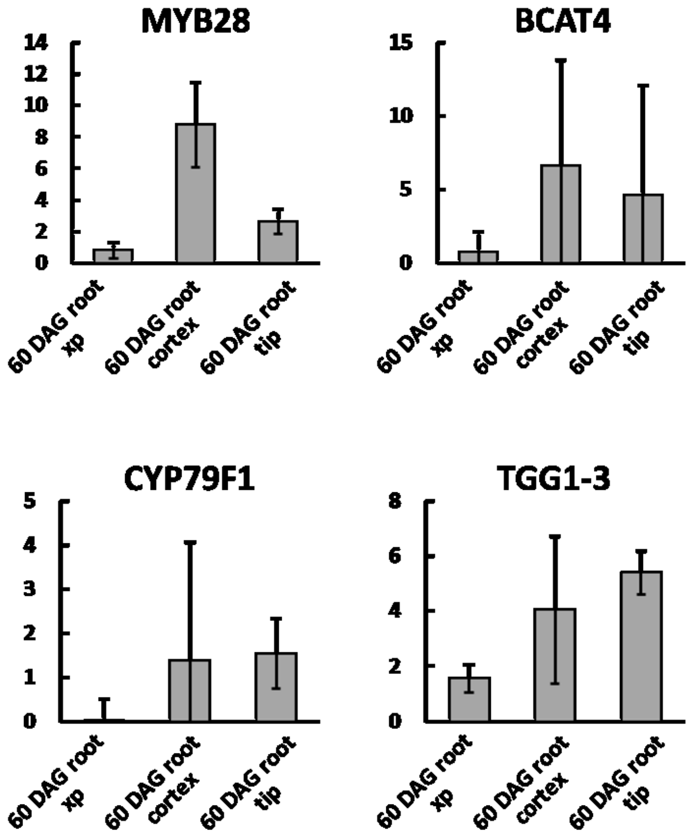

Supplementary Figure S15. The relative expression level of the four pungency-related genes estimated from RT-qPCR. The expression levels were adjusted by two reference genes (Actin and CAM7). The expression value of each sample is relative to those of MYB28 of 7 DAG roots. The values are mean ± SD (n = 3).

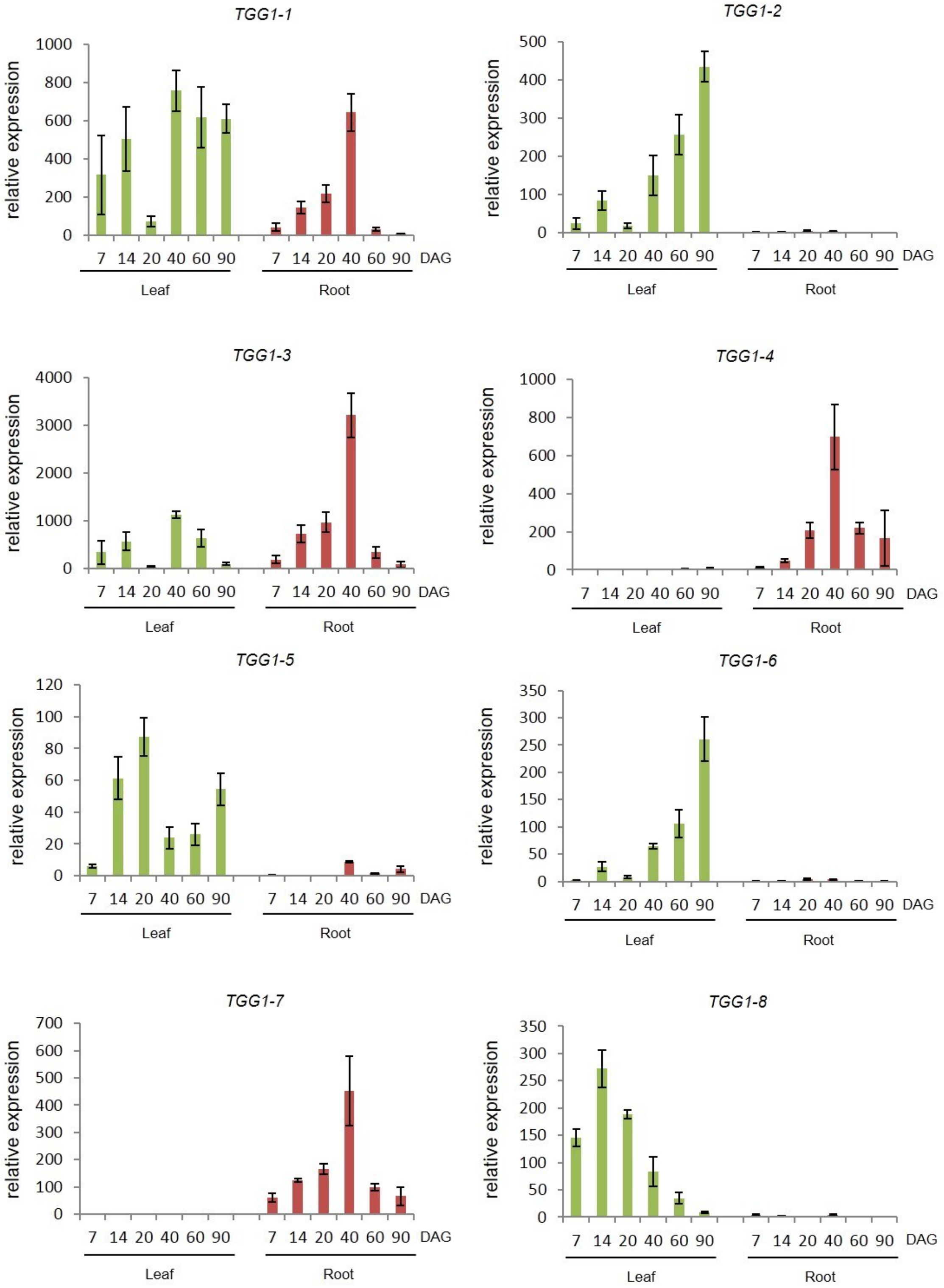
TGG1-1
relative expression
1000
800
600
400
200
0
7 14 20 40 60 90
7 14 20 40 60 90 DAG
Leaf
Root
TGG1-2
relative expression
500
400
300
200
100
0
7 14 20 40 60 90
7 14 20 40 60 90 DAG
Leaf
Root
TGG1-3
relative expression
4000
3000
2000
1000
0
7 14 20 40 60 90
7 14 20 40 60 90 DAG
Leaf
Root
TGG1-4
relative expression
1000
800
600
400
200
0
7 14 20 40 60 90
7 14 20 40 60 90 DAG
Leaf
Root
TGG1-5
relative expression
120
100
80
60
40
20
0
7 14 20 40 60 90
7 14 20 40 60 90 DAG
Leaf
Root
TGG1-6
relative expression
350
300
250
200
150
100
50
0
7 14 20 40 60 90
7 14 20 40 60 90 DAG
Leaf
Root
TGG1-7
relative expression
700
600
500
400
300
200
100
0
7 14 20 40 60 90
7 14 20 40 60 90 DAG
Leaf
Root
TGG1-8
relative expression
350
300
250
200
150
100
50
0
7 14 20 40 60 90
7 14 20 40 60 90 DAG
Leaf
Root

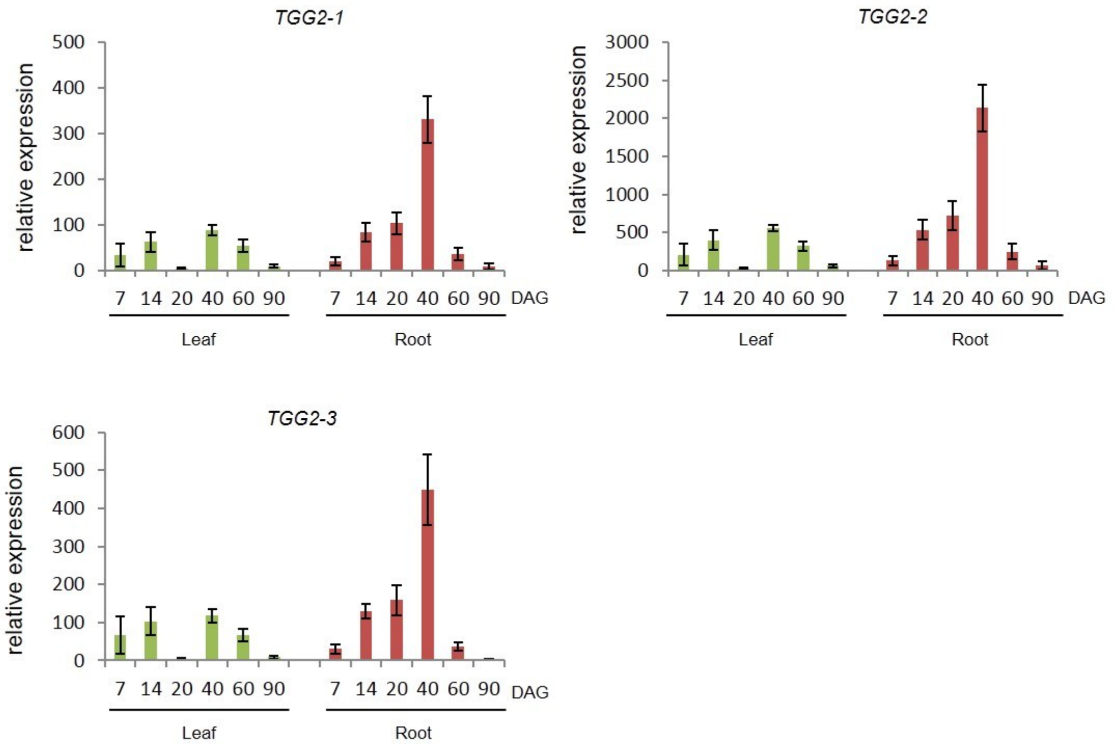

Supplementary Figure S16. The relative expression level of myrosinase genes in leaf and root. The expression levels in leaf and root tip were analyzed by RNA-seq analysis. The values are mean ± SE (n = 3).

Supplementary Table S1. NCBI UniGene dataset of *R. sativus* used for the scaffold quality evaluation.

Species	No. sequences	Description
R.sativus convar. *oleifera*	5,652	Arena oil type. Michigan State University, East Lansing
R.sativus	5,381	Early Sarlet Globe, NK Lawn & Garden
R.sativus	6,021	Rat-Tail Radish #3870, John Scheepers Kichen Garden Seeds
R.sativus	127	Other

Supplementary Table S2. Number of radish Unigenes mapped to Scaffolds (≥ 500 bp).

	Hit region coverage %				
	> 0	≥ 50	≥ 70	≥ 90	100
No. of mapped Unigenes	16,911	16,438	15,773	13,571	39
Mapped Unigenes / total Unigenes* (%)	98.43	95.68	91.80	78.99	0.23

*A total 17,181 Unigenes from NICBI UniGene

Supplementary Table S3. Estimation of genome size of *R. sativus* based on flow cytometry.

Sample	*R. sativus* 2C peak position (median)	*A. thaliana* 4C peak position (median)	Size (Mb)
1	87	48	569.1
2	85	46	580.2
3	84	46	573.4
Average			574.2

Supplementary Table S4. Sequencing and assembly of a cDNA library derived from leaves of 20-day old seedlings of *R. sativus*.

Library name	20-days_leaves_1
Insert size	300 bp
No. of entries	43,023,361
No. of bases	8,604,672,200
No. of contigs	99,096
No. of ORFs	1,642,042
No. of longest ORFs	53,846

The sequence data were registered as BioProject ID PRJDB707.

Supplementary Table S5. Analysis of gene models and mRNA.

	Scaffold ≥ 500 b				
Hit region coverage %	> 0	≥ 50	≥ 70	≥ 90	100
Number of hit genes	50,196	22,949	16,380	9,755	5,554
Number of hit mRNA / number of mRNA %	93.22	42.62	30.42	18.12	10.31
Number of hit gene model / number of gene model %	93.83	42.90	30.62	18.24	10.38

Supplementary Table S6. Gene ontology for *R. sativus*, *A. thaliana*, and *B. rapa*.

	GO ID		*R.sativus %*	*A.thaliana %*	*B.rapa %*
molecular_function GO:0003674	GO:0016209	antioxidant activity	0.335	0.361	0.388
	GO:0005488	binding	51.328	49.043	47.953
	GO:0003824	catalytic activity	37.977	39.322	39.575
	GO:0016247	channel regulator activity	0.012	0.020	0.023
	GO:0009055	electron carrier activity	1.014	1.168	1.281
	GO:0030234	enzyme regulator activity	0.772	0.756	0.875
	GO:0016530	metallochaperone activity	0.005	0.009	0.004
	GO:0060089	molecular transducer activity	0.455	0.608	0.524
	GO:0044092	negative regulation of molecular function	0.584	0.533	0.630
	GO:0001071	nucleic acid binding transcription factor activity	1.621	1.633	1.928
	GO:0045735	nutrient reservoir activity	0.098	0.128	0.116
	GO:0044093	positive regulation of molecular function	0.150	0.218	0.189
	GO:0000988	protein binding transcription factor activity	0.090	0.117	0.104
	GO:0004872	receptor activity	0.127	0.181	0.145
	GO:0065009	regulation of molecular function	1.124	1.216	1.294
	GO:0005198	structural molecule activity	1.247	1.346	1.428
	GO:0005215	transporter activity	3.060	3.342	3.543
cellular_component GO:0005575	GO:0005623	cell	24.886	24.918	24.645
	GO:0044464	cell part	24.886	24.918	24.645
	GO:0031012	extracellular matrix	0.030	0.024	0.030
	GO:0005576	extracellular region	0.863	0.825	0.968
	GO:0044421	extracellular region part	0.026	0.014	0.039
	GO:0032991	macromolecular complex	8.898	8.783	8.972
	GO:0016020	membrane	15.533	15.316	15.828
	GO:0044425	membrane part	7.511	7.588	7.716
	GO:0031974	membrane-enclosed lumen	0.672	0.820	0.731
	GO:0009295	nucleoid	0.009		
	GO:0043226	organelle	12.794	12.682	12.486
	GO:0044422	organelle part	3.892	4.101	3.931
	GO:0019012	virion		0.005	
	GO:0044423	virion part		0.005	
	GO:0045202	synapse			0.004
	GO:0044456	synapse part			0.004
biological_process GO:0008150	GO:0022610	biological adhesion	0.003	0.009	0.004
	GO:0065007	biological regulation	5.060	5.330	5.581
	GO:0071840	cellular component organization or biogenesis	0.945	1.062	1.019
	GO:0009987	cellular process	25.564	24.143	24.446
	GO:0032502	developmental process	0.043	0.038	0.046
	GO:0051234	establishment of localization	5.399	5.778	5.986
	GO:0002376	immune system process	0.004	0.004	0.002
	GO:0051179	localization	5.429	5.808	6.019
	GO:0040011	locomotion	0.002		
	GO:0008152	metabolic process	41.455	40.166	39.422
	GO:0051704	multi-organism process	0.079	0.063	0.081
	GO:0032501	multicellular organismal process	0.110	0.093	0.115
	GO:0048519	negative regulation of biological process	0.055	0.067	0.060
	GO:0048518	positive regulation of biological process	0.072	0.085	0.076
	GO:0050789	regulation of biological process	5.009	5.277	5.529
	GO:0000003	reproduction	0.084	0.069	0.090
	GO:0022414	reproductive process	0.071	0.053	0.071
	GO:0050896	response to stimulus	2.409	2.794	2.519
	GO:0023052	signaling	1.248	1.485	1.317
	GO:0044699	single-organism process	6.959	7.675	7.617

Supplementary Table S7. Summary of gene family clustering*.

Species	No. of genes	No. of genes after cdhit	No. of groups	No. of genes in families	No. of groups specific	No. of RBHs / *R.sativus*
R.sativus	64,657	57,275	21,339	41,612	1,272	-
A.lyrata	32,670	29,961	19,473	24,195	426	18,684 (57.1%)
A.thaliana	34,407	27,296	19,418	24,084	137	19,008 (55.2%)
B.rapa	41,173	36,369	20,435	28,767	250	25,913 (62.9%)
C.papaya	27,793	26,659	11,878	16,407	668	11,614 (41.8%)
Total	200,700	177,560	92,543	135,065		

*Description of clusters of orthologous (or paralogous) genes was obtained after applying OrthoMCL. We report also the number of *R.sativus* genes with a reciprocal best-hit (RBH) [11] relationship with the organism. Numbers in parentheses indicate the percent of RBH with *R. sativus*.

Supplementary Table S8. Pseudomolecules with and without gaps for the 9 linkage groups.

	pseudomolecule [bp]	pseudomolecule without gap [bp]
LN1	14,922,660	11,308,404
LN2	26,577,246	19,955,032
LN3	27,489,656	21,360,322
LN4	25,091,076	19,126,438
LN5	23,622,480	18,728,960
LN6	15,420,204	11,294,379
LN7	15,468,881	11,524,955
LN8	15,634,024	11,695,520
LN9	15,611,764	12,171,734
Total	179,837,991	137,165,744

Supplementary Table S9. Summary of the transcript reads of *R. sativus* var. *hortensis* cv. Aokubi.

Description	Yield (Mbases)	SD	# Reads (Million)	SD	% of >= Q30 Bases (PF)	SD	Mean Quality Score (PF)	SD
Day 7 root	2889	125	29	1	88	0	35	0
Day 14 root	2854	157	29	2	88	0	35	0
Day 20 root	2365	508	24	5	87	0	34	0
Day 40 root vascular cambium	2990	456	32	4	88	0	35	0
Day 40 root xylem parenchyma	3032	406	34	1	88	0	35	0
Day 40 root cortex	2463	241	30	2	88	0	35	0
Day 60 root vascular cambium	3402	1416	34	14	89	2	35	0
Day 60 root xylem parenchyma	3162	1458	32	15	89	2	35	0
Day 60 root cortex	2754	701	28	7	88	2	35	0
Day 7 leaf	2888	127	29	1	88	0	35	0
Day 14 leaf	2959	242	30	2	88	1	35	0
Day 20 leaf	2868	564	29	6	86	0	34	0
Day 40 leaf	3545	166	31	6	88	2	35	0
Day 60 leaf	3751	2465	38	25	88	1	35	0
Mean	2995	645	30	6	88	1	35	0

Supplementary Table S10. Significant GOs for each of the cluster generated from SOM analyses. Number of annotated genes were 40,705. BP: Biological Process, CC: Cellular Component, MF: Molecular Function.

Excel file

Supplementary Table S11. Significant KEGG pathways found in the cluster of upregulated genes at several developmental stages and tissues.

Excel file

Supplimentary Table S12 Gene inventory of the glucosinolate biosynthesis and the degradation pathway in *R. sativas*

Gene ID	AGI code	Name	Number
RSG13682.t1	AT3G19710.1	BCAT4	1
RSG45676.t1	AT3G49680.1	BCAT3	2
RSG51233.t1	AT3G49680.1		
RSG11180.t1	AT5G23010.1	MAM1	3
RSG13852.t1	AT5G23010.1		
RSG24637.t1	AT5G23010.1		
RSG39553.t1	AT4G13430.1	IPMI LSU1	1
RSG05959.t1	AT2G43100.1	IPMI SSU2	1
RSG14423.t1	AT5G14200.1	IPMDH1	1
RSG07720.t1	AT1G16410.1	CYP79F1	1
RSG12342.t1	AT4G13770.1	CYP83A1	1
RSG37909.t1	AT3G03190.1	GSTF11	1
RSG28592.t1	AT1G78370.1	GSTF20	1
RSG01617.t1	AT4G30530.1	GGP1	3
RSG28117.t1	AT4G30530.1		
RSG30252.t1	AT4G30530.1		
RSG38701.t1	AT2G20610.1	SUR1	1
RSG35660.t1	AT1G24100.1	UGT74B1	1
RSG38352.t1	AT2G31790.1	UGT74C1	2
RSG13253.t1	AT2G31790.1		
RSG03557.t1	AT1G18590.1	SOT17	2
RSG12634.t1	AT1G18590.1		
RSG07388.t1	AT1G74090.1	SOT18	9
RSG12630.t1	AT1G74090.1		
RSG12631.t1	AT1G74090.1		
RSG12632.t1	AT1G74090.1		
RSG12635.t1	AT1G74090.1		
RSG32297.t1	AT1G74090.1		
RSG32298.t1	AT1G74090.1		
RSG49978.t1	AT1G74090.1		
RSG34220.t1	AT1G74090.1		
RSG02956.t1	AT2G14750.1	APK1	1
RSG17909.t1	AT4G39940.1	APK2	3
RSG23430.t1	AT4G39940.1		
RSG33797.t1	AT4G39940.1		
RSG05041.t1	AT5G27380.1	GSH2	2
RSG17778.t1	AT5G27380.1	GSH2	
RSG00914.t1	AT4G23100.3	GSH1	3
RSG00915.t1	AT4G23100.3	GSH1	
RSG07930.t1	AT4G23100.3	GSH1	
RSG43277.t1	AT5G54810.1	TSB1	1

Supplementary Table S12. Continued.

RSG02731.t1	AT5G05260.1	CYP79A2	3
RSG08366.t1	AT5G05260.1		
RSG38144.t1	AT5G05260.1		
RSG17908.t1	AT4G39950.1	CYP79B2	3
RSG23431.t1	AT4G39950.1		
RSG33798.t1	AT4G39950.1		
RSG34152.t1	AT2G22330.1	CYP79B3	1
RSG48186.t1	AT4G31500.1	CYP83B1	1
RSG18947.t1	AT2G30860.1	GSTF9	1
RSG32253.t1	AT2G30860.1	GSTF9	
RSG32254.t1	AT2G30870.1	GSTF10	1
RSG07387.t1	AT1G74100.1	SOT16	2
RSG28254.t1	AT1G74100.1	SOT16	
RSG43366.t1	AT4G12030.2	BAT5	1
RSG15203.t1	AT1G21100.1	IGMT1	1
RSG26550.t1	AT1G21100.1	IGMT1	
RSG59991.t1	AT1G21100.1	IGMT1	
RSG15204.t1	AT1G21120.1	IGMT2	1
RSG32181.t1	AT1G65880.1	BZO1	1
RSG00041.t1	AT2G25450.1	GS-OH	1
RSG51376.t1	AT1G65860.1	FMO GS-OX1	2
RSG57085.t1	AT1G65860.1		
RSG57158.t1	AT1G62540.1	FMO GS-OX2	2
RSG15645.t1	AT1G62540.1		
RSG14855.t1	AT1G12140.1	FMO GS-OX5	2
RSG28761.t1	AT1G12140.1		
RSG11506.t1	AT5G57220.1	CYP81F2	3
RSG29212.t1	AT5G57220.1		
RSG29215.t1	AT5G57220.1		
RSG40296.t1	AT5G57220.1	CYP81F2	1
RSG10178.t1	AT4G37400.1	CYP81F3	2
RSG23271.t1	AT4G37400.1		
RSG10180.t1	AT4G37410.1	CYP81F4	1
RSG16088.t1	AT5G61420.2	MYB28	3
RSG23384.t1	AT5G61420.2	MYB28	
RSG53581.t1	AT5G61420.1	MYB28	
RSG00789.t1	AT5G07690.1	MYB29	2
RSG09585.t1	AT5G07690.1	MYB29	
RSG23127.t1	AT5G26000.1	TGG1	8
RSG25390.t1	AT5G26000.1		
RSG30299.t1	AT5G26000.1		
RSG33328.t1	AT5G26000.1		
RSG36879.t1	AT5G26000.1		
RSG40666.t1	AT5G26000.1		
RSG48132.t1	AT5G26000.1		
RSG52100.t1	AT5G26000.1		
RSG30297.t1	AT5G25980.3	TGG2	3
RSG30298.t1	AT5G25980.3		
RSG40189.t1	AT5G25980.3		

Supplementary Table S13. Summary of entries, bases, sequence coverage, clone coverage, insert size for each library.

Library name	Insert size [bp]	No. of entries	No. of bases [bp]	Sequence coverage	Sequencer
PE300_L001	300	123,214,198	24,642,839,600	42.9	Illumina Hiseq2000
PE300_L002	300	134,426,349	26,885,269,800	46.8	
PE500_L005	500	28,689,277	5,737,855,400	10.0	
MP8K_L006	8,000	7,310,349	1,462,069,800	2.5	
MP8K_L007	8,000	8,808,233	1,761,646,600	3.1	
MK20K_L006	20,000	6,706,289	1,341,257,800	2.3	
MK20K_L007	20,000	8,062,897	1,612,579,400	2.8	
MK40K_L006	40,000	3,211,854	642,360,800	1.1	
MK40K_L007	40,000	3,884,995	776,999,000	1.4	
SE	-	13,743,860	5,040,510,672	8.8	Roche FLX titunium
Total		338,058,301	69,903,388,872	121.8	

Supplementary Table S14. Assembly results summary by Ray, CLC bio, and Newbler; “input” means used file’s property with sequencer type, read type, and insert size; “contig” means number of contig, number of bases (Mb), average contig size (bp), N50 contig size (bp), and largest contig size (bp); “scaffold” means number of scaffold, number of bases (Mb), average scaffold size (bp), N50 scaffold size (bp), and largest scaffold size(bp).

			INPUT						CONTIG					SCAFFOLD				
case	assembler	version	454(x10)	PE300	PE500	MP8K	MP20K	MP40K	number Of Contig	number Of Base [Mb]	avg Contig Size [bp]	N50 Contig Size [bp]	largest Contig Size [bp]	number Of Scaffold	number Of Bases [Mb]	avg Scaffold Size [bp]	N50 Scaffold Size [bp]	largest Scaffold Size [bp]
1	Ray	2.0.0	1	1	1				144,609	280.47	1,939	6,341	136,286	144,147	281.73	1,954	6,429	136,286
2	CLC GenomeWorkbench	5.5.1	1	1	1	1			176,279	268.53	1,523	5,137	85,323	142,484	302.41	2,122	9,320	107,236
3	newbler	2.7	1	1	1	1			75,208	261.10	3,471	6,760	66,146	9,013	284.88	31,607	105,385	942,732
3.1	newbler+SSPACE	2.7+2.0	1	1	1	1	1	1	-	-	-	-	-	40,123	353.77	40,728	158,632	1,313,578

Supplementary Table S15. Repeat sequences obtained from analysis REPET.

Class	Subclass	Length [Mb]	Length/ Scaffold %
I	DIRS	0.04	0.01
	LTR	41.65	10.85
	LINE	15.92	4.15
	SINE	2.62	0.68
	noCat	2.88	0.75
II	Helitron	2.81	0.73
	Maverick	0.23	0.06
	MITE	0.94	0.25
	TIR	9.74	2.54
	noCat	0.45	0.12
NA	PotentialHostGene	19.55	5.09
noCat	Helitron|LARD	0.15	0.04
noCat	SINE|TIR	0.11	0.03
noCat	noCat	36.24	9.44
Satellite		1.46	0.38
Simple_repeat		1.36	0.35
Low_complexity		4.41	1.15
Total		140.52	36.61

Supplementary Table S16. Marker information used to construct a linkage map.

Excel file

Supplementary Table S17. Relationships among linkage groups. Blue line indicates same direction and red line indicates reverse direction.

Present research	LN1	LN2	LN3	LN4	LN5	LN6	LN7	LN8	LN9
Number of markers	131	176	217	212	202	116	124	102	96
Kazusa LN	LN1+	LN2+	LN3+	LN4+	LN5+	LN6+	LN7+	LN8+	LN9+
	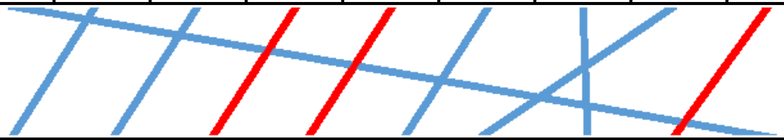								
Tohoku LN	LN2+	LN3+	LN4-	LN5-	LN6+	LN8+	LN7+	LN9-	LN1+

Supplementary Table S18. Sequence of primer pairs used for RT-qPCR for sucrose metabolism and pungency related genes.

Gene		Primer name	Sequence (5' - 3')	Source
RsSUS1a	Forward	RsSUS1a_1L	GGTAAAGGCATCCTCCAACAG	Developed for this study
	Reverse	RsSUS1a_1R	CATGGTGGCAACACAATAGC	Developed for this study
RsSUS1b	Forward	RsSUS1b_1L	GAAACGAAGTCCTTGCCTTG	Developed for this study
	Reverse	RsSUS1b_1R	CCCATGGTGGCAACACTATT	Developed for this study
RsMYB28a	Forward	RsMYB28a_1F	GCGAGTTTAGTTCGGAGGAG	Developed for this study
	Reverse	RsMYB28a_1R	TGCATCTAGGGAATGCAAGC	Developed for this study
RsBCAT4	Forward	RsBCAT4_Fwd	AACGCTCTCCGCCTTCAGTC	Developed for this study
	Reverse	RsBCAT4_Rev	CCGAGGGACAAGGCATACA	Developed for this study
RsCYP79F1	Forward	RsCYP79F1_Fwd	CGGCAATTTACCCGAACTAA	Developed for this study
	Reverse	RsCYP79F1_Rev	GTTTATGACGATGGCGTGTG	Developed for this study
RsTGG1	Forward	RsTGG1c_Fwd	ATTCCACGCGGATTGACTAC	Developed for this study
	Reverse	RsTGG1c_Rev	GACGGTAAATCCTTGGCAGA	Developed for this study
RsCAM7	Forward	RsCAM7_1L	TGACCGATGACCAGATCTCA	Developed for this study
	Reverse	RsCAM7_1R	AGTGACCTCATCACGGTTCC	Developed for this study
Actin	Forward		ATCAGGAAGGACTTGTACGGTAAC	Zou et al.[76]
	Reverse		GCTGAGGGAAGCAAGAATGGAACC	Zou et al.[76]
